# SIPA1 Enhances Aerobic Glycolysis Through HIF-2α Pathway to Promote Breast Cancer Metastasis

**DOI:** 10.3389/fcell.2021.779169

**Published:** 2022-01-12

**Authors:** Chenguang Yao, Jun Weng, Lingyun Feng, Wanjun Zhang, Yan Xu, Peijing Zhang, Yoshimasa Tanaka, Li Su

**Affiliations:** ^1^ Key Laboratory of Molecular Biophysics of Ministry of Education, College of Life Science and Technology, Huazhong University of Science and Technology, Wuhan, China; ^2^ Medical Innovation Center, Graduate School of Medicine, Kyoto University, Kyoto, Japan; ^3^ Center for Medical Innovation, Nagasaki University, Nagasaki, Japan

**Keywords:** aerobic glycolysis, breast cancer, EPAS1, HIF-2α, signal-induced proliferation-associated 1

## Abstract

Increased dependence on aerobic glycolysis is characteristic of most cancer cells, whereas the mechanism underlying the promotion of aerobic glycolysis in metastatic breast cancer cells under ambient oxygen has not been well understood. Here, we demonstrated that aberrant expression of signal-induced proliferation-associated 1 (SIPA1) enhanced aerobic glycolysis and altered the main source of ATP production from oxidative phosphorylation to glycolysis in breast cancer cells. We revealed that SIPA1 promoted the transcription of *EPAS1*, which is known as the gene encoding hypoxia-inducible factor-2α (HIF-2α) and up-regulated the expression of multiple glycolysis-related genes to increase aerobic glycolysis. We also found that blocking aerobic glycolysis by either knocking down SIPA1 expression or oxamate treatment led to the suppression of tumor metastasis of breast cancer cells both *in vitro* and *in vivo*. Taken together, aberrant expression of SIPA1 resulted in the alteration of glucose metabolism from oxidative phosphorylation to aerobic glycolysis even at ambient oxygen levels, which might aggravate the malignancy of breast cancer cells. The present findings indicate a potential target for the development of therapeutics against breast cancers with dysregulated SIPA1 expression.

## Introduction

Cancer cells rely on aerobic glycolysis for energy resources, even under the condition of ambient oxygen levels (Hanahan and Weinberg, 2011). This aberrant metabolic process is of great benefit for rapid energy production and increased levels of intermediates for other metabolic pathways, and continuously yields acidic lactate altering microenvironment, impairing tumor immune responses and stromal cell integrity, and ultimately leading to tumor progression (Kroemer and Pouyssegur, 2008; Simoes et al., 2015; Garcia-Canaveras et al., 2019). Although aerobic glycolysis is characteristic for some cancer cells, the mechanism underlying the conversion of metabolic pathway of aerobic glycolysis has not been fully elucidated yet.

Breast cancer is a heterogeneous disease characterized by metabolic rewriting from oxidative phosphorylation to aerobic glycolysis in a specific malignant mass, whereas functional mitochondria is pivotal for the survival of cancer cells (Jeon et al., 2013; Lanning et al., 2017; Wang et al., 2020). Hypoxia-inducible factor-1 (HIF-1α) is an essential regulatory factor for aerobic glycolysis and other neoplastic bioprocesses in cancer cells in both hypoxic as well as oxygenated regions of tumors (Denko, 2008). By inducing glycolysis-related genes products such as hexokinase 2 (HK2), lactate dehydrogenase A (LDHA), and glucose transporter 1 (GLUT1, also known as solute carrier family A1, SLC2A1), HIF-1α switches the glucose metabolism of hypoxic tumor cells to the glycolytic pathway. This metabolic switch might cause a shift in energy production in cancer cells (Denko, 2008). Whereas HIF-1α was initially identified as a key factor in response to hypoxia, accumulating evidence has revealed that HIF-1α could be also regulated by hypoxia-independent pathways such as oncogene activation or loss of tumor suppressors, which links metabolic reprogramming to tumorigenesis and cancer metastasis (Denko, 2008; Rankin and Giaccia, 2016; Zhang et al., 2019). As one of the oncogenic transcriptional factors regulating the glycolytic phenotype of breast cancer, c-myc can drive glycolytic programming by directly up-regulating the transcription of glycolysis-related genes including GLUTs, HK2, and LDHA (Hsieh et al., 2015). In addition, p53 and K-ras were demonstrated to be involved in the regulation of glycolysis in hepatocarcinoma (Kawada et al., 2017; Kim et al., 2019). Moreover, HIF-2α, which is encoded by *EPAS1* gene, having 48% homology to HIF-1α at an amino acid sequence, is expressed in many tumor cells and facilitates aerobic glycolysis by targeting and transcriptionally activating glycolysis-related genes (Warnecke et al., 2004; Kim et al., 2009; Chen et al., 2017), and promotes breast cancer cell epithelial-mesenchymal transition (EMT) and invasion (Yang et al., 2019). It has been reported that HIF-2α activates c-myc in a way that mimics a response to hypoxia and then triggers the expression of glutamine transporter ASCT2 and of glutaminase 1 (GLS1), resulting in an increase in glutamine uptake and catabolism in SiHa and HeLa cervical carcinoma cells (Perez-Escuredo et al., 2016). Although these regulators have been shown to be responsible for aerobic glycolysis, the precise mechanism how breast cancer cells switch this metabolic process has not been well delineated yet.

Signal-induced proliferation-associated 1 (SIPA1), a member of Rap1GAP family (Wada et al., 1997), is aberrantly expressed in breast cancer, colorectal cancer, melanoma and prostate cancer cells, participating in the regulation of tumor cell proliferation, adhesion, invasion and metastasis (Shimizu et al., 2011; Ji et al., 2012; Mathieu et al., 2012; Zhang et al., 2015). Recent studies confirmed that SIPA1 interacted with promoters of integrin subunit β1 (*ITGB1*) and cluster of differentiation 44 (*CD44*) and activated their transcription (Zhang et al., 2015; Wang et al., 2020). It is, however, not clear whether SIPA1 could regulate or alter the metabolism in breast cancer cells.

In the present study, we set out to clarify the effect of aberrant expression of SIPA1 on glucose metabolism in breast cancer cells and demonstrated that SIPA1 enhanced aerobic glycolytic flux and up-regulated the expression of glycolysis-related genes in breast cancer cells through transcriptionally activating HIF-2α. The SIPA1/HIF-2α axis shifted the ATP source from conventional oxidative phosphorylation to aerobic glycolysis, even in the presence of functional mitochondria, and promoted breast cancer invasion and metastasis both *in vitro* and *in vivo*. The findings strongly suggested that the SIPA1/HIF-2α axis is critical for aerobic glycolysis and pivotal for metastasis in breast cancers highly expressing SIPA1.

## Results

### SIPA1 Modulates Aerobic Glycolysis in Breast Cancer Cells

**FIGURE 1:**
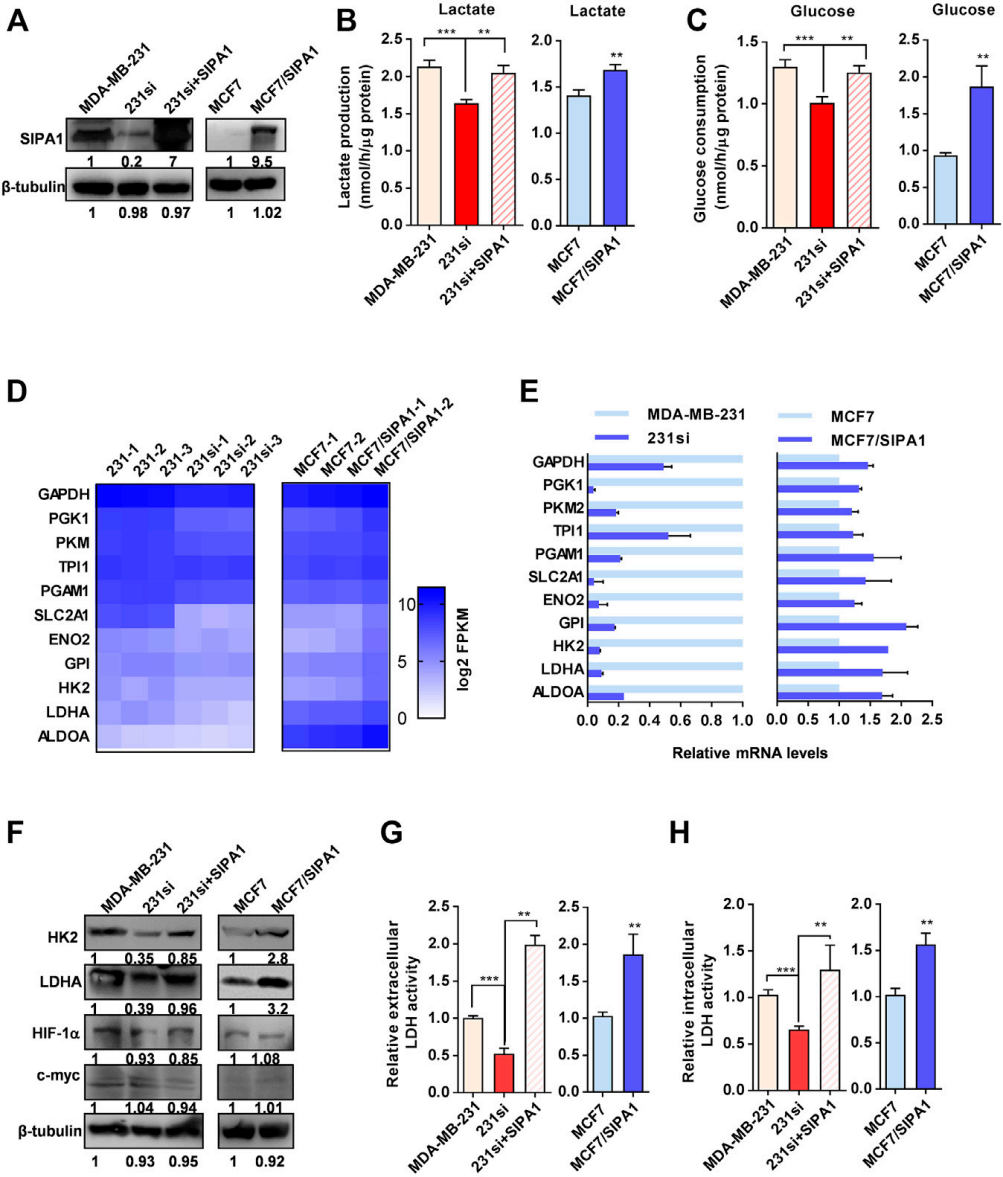
SIPA1 enhances aerobic glycolysis in breast cancer cells under a normoxic condition. **(A)** SIPA1 protein expression levels in breast cancer cells were determined by Western blotting analysis. 231si (SIPA1-knocked down MDA-MB-231); 231si + SIPA1 (SIPA1-resumed 231si); MCF7/SIPA1 (SIPA1 overexpressed MCF7). β-tubulin was included as a loading control. **(B–C)** Effect of SIPA1 on the lactate production **(B)** and the glucose consumption **(C)** in breast cancer cell lines. **(D)** Heatmap of mRNA levels of major glycolysis-related genes in three independent parental and SIPA1-knockdown MDA-MB-231 cells, and two independent MCF7 and MCF/SIPA1 cells were presented with Log_2_ FPKM by transcriptome sequencing and analysis. **(E)** mRNA levels of glycolysis-related genes were determined by qRT-PCR. Data were shown as mean ± s.d. The experiments were conducted in triplicate. Mitochondrial succinate dehydrogenase cytochrome b560 subunit (SDHC) was included as an endogenous control. **(F)** Expression of HK2, LDHA, HIF-1α and c-myc were determined by Western blotting. β-tubulin was included as a control. **(G–H)** the extracellular lactate dehydrogenase (LDH) activity **(G)** and intracellular LDH activity **(H)** were determined. Data are shown as mean ± s.d triplicate measuements (n = 3). **p < 0.01, ***p < 0.001. (Student^’^s *t*-test).

To examine the status of aerobic glycolysis in metastatic breast cancer cells with ambient oxygen, we first determined the lactate production and glucose consumption in four different breast cancer cell lines. As shown in Supplementary Figure S1A, three triple-negative breast cancer (TNBC) cell lines, SUM159, MDA-MB-231 and BT549, all with high level of SIPA1 expression, consumed more glucose and produced more lactate than MCF7, a non-TNBC cell line with low SIPA1 expression (Supplementary Figures S1A–S1C), indicating that a high level of SIPA1 expression might be responsible for high aerobic glycolysis in breast cancer cells. To verify the correlation between SIPA1 and aerobic glycolysis, we established MDA-MB-231 and BT549 cell lines expressing a low level of SIPA1 and an MCF7 cell line overexpressing a high level of SIPA1 (MCF7/SIPA1) (Figure 1A and Supplementary Figure S2A), and we found knocking down SIPA1 decreased the levels of lactate production and glucose consumption, whereas forced expression of SIPA1 exhibited the opposite effects in these cells (Figures 1B,C and Supplementary Figures S2B–C). Furthermore, we examined the transcriptional levels of glycolysis-related genes of parental and SIPA1-knockdown MDA-MB-231 cells, as well as those of parental and SIPA1-overexpressing MCF7 cells by RNA sequencing. As shown in Figure 1D, knocking down SIPA1 down-regulated mRNA levels of nearly all glycolysis-related genes in MDA-MB-231 cells, whereas overexpressing SIPA1 up-regulated them in MCF7 cells. It was then confirmed by qRT-PCR assay that SIPA1 modulated the transcription levels of the glycolysis-related genes in MDA-MB-231, BT549 and MCF7 cells (Figure 1E and Supplementary Figure S3). The expression of key enzymes HK2 and LDHA was detected by Western blotting and we confirmed that knocking down SIPA1 significantly down-modulated the expression of HK2 and LDHA in MDA-MB-231 cells, whereas overexpressing SIPA1 up-regulated them in MCF7 cells (Figure 1F). Since LDHA is a critical enzyme for the conversion of pyruvate to lactate at the final step of aerobic glycolysis, we set out to determine whether LDH activity was modulated by SIPA1 in breast cancer cells. As shown in Figures 1G,H, knocking down SIPA1 in MDA-MB-231 cells significantly decreased both extracellular and intracellular LDH activities, whereas forced-expression of SIPA1 in 231si cells increased both LDH activities. In MCF7 cells, overexpressing SIPA1 also greatly increased both LDH activities (Figures 1G,H).

Additionally, it is worthy of note that HIF-1α and c-myc have been reported to serve as dominant regulators of glycolysis by targeting multiple glycolysis-related genes in a variety of cancers (Gatenby and Gillies, 2004). The expression levels of HIF-1α and c-myc were, however, not affected by SIPA1 both in MDA-MB-231 and MCF7 cells (Figure 1C). Taken together, SIPA1 might be a modulator of aerobic glycolysis under normoxia in breast cancer cells.

### SIPA1 Alters ATP Production Source in Breast Cancer Cells

**FIGURE 2:**
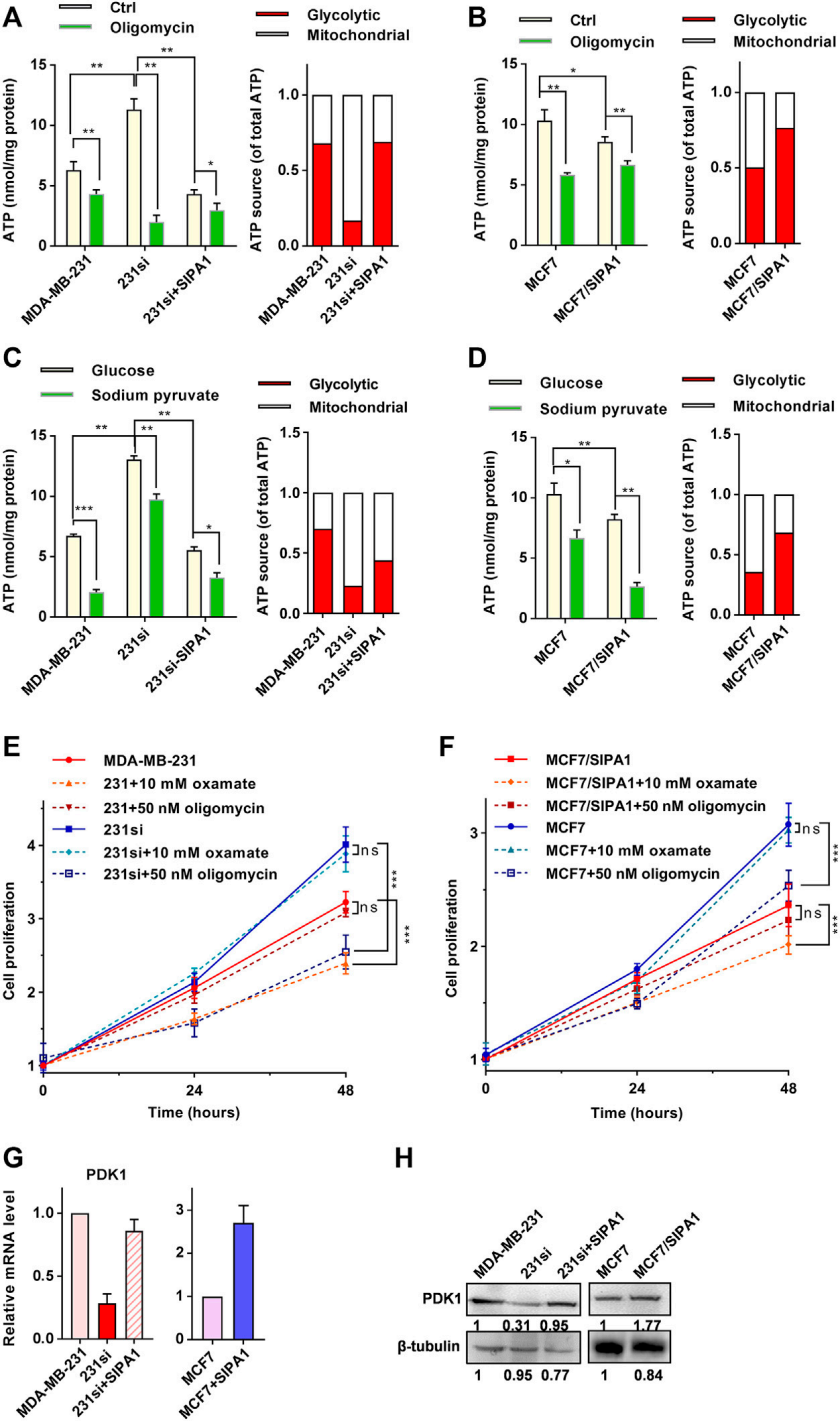
SIPA1 alters ATP production source from respiration to aerobic glycolysis. **(A–B)** ATP levels in MDA-MB-231 **(A)** and MCF7 **(B)** with various SIPA1 expression levels after treatment with oligomycin or not. A mitochondria ATP level was calculated by the following formula (ATP_(m)_ = ATP_(T)_ - ATP_(g)_), in which total ATP level (ATP_(T)_) was measured after incubating cells with 15 mM glucose and the glycolytic ATP (ATP_(g)_) were measured after incubating cells with 15 mM glucose and 100 nM oligomycin for 5 h. Yellow bars: control; green bars: oligomycin (left panel). Based on the left panel, the contribution of each ATP source on cellular ATP production was estimated. Red: aerobic glycolysis; white: mitochondrial respiration (right panel). **(C–D)** Effect of sodium pyruvate as a sole carbon source on ATP production in MDA-MB-231 **(C)** and MCF7 cells **(D)** with various SIPA1 expression levels. Cells were grown in the medium containing glucose or sodium pyruvate as a sole carbon source, and the cellular ATP level was measured. Yellow bars, glucose; green bars, sodium pyruvate (left panel). Based on the left panel, the contribution of each ATP source on cellular ATP production was estimated. Red: aerobic glycolysis; white: mitochondrial respiration (right panel). **(E–F)** Effect of oxamate and oligomycin on the proliferation of MDA-MB-231 **(E)** and MCF7 cells **(F)**. **(G)** mRNA levels of PDK1 were determined by qRT-PCR. **(H)** Protein levels of PDK1 were determined by Western blotting. Data were shown as mean ± s.d of triplicate measurements (n = 3). *p < 0.05, **p < 0.01, ***p < 0.001, ns, no significance. (Student^’^s *t*-test).

When breast cancer cells were incubated with glucose as an exclusive carbon source, as shown in Figure 2A (left panel), the cellular ATP level was increased by knocking down SIPA1 in MDA-MB-231 and restored by forced expression of SIPA1 in 231si cells. A decrease in the level of ATP was also observed in MCF7 cells overexpressing SIPA1 (Figure 2B, left).

Since overexpressing SIPA1 in breast cancer cell lines increased glucose consumption and lactate production (Figures 1D,E), but decreased ATP production (Figures 2A,B), it is likely that there might exist a SIPA1-induced energy production switch from respiration to fermentation in breast cancer cells. To address this hypothesis, we treated cells with the mitochondrial ATPase inhibitor oligomycin, and found that 100 nM oligomycin treatment decreased ATP levels in breast cancer cells (Figures 2A,B, left panel). Notably, the ATP level in 231si cells was significantly decreased to one-sixth of its original value after oligomycin treatment, suggesting that a significant amount of ATP (about 80%) was produced by respiratory pathway in 231si cells, whereas as much as 70% ATP were produced *via* aerobic glycolysis in MDA-MB-231 cells or 231si cells with forced-expression of SIPA1 (Figure 2A, right panel). Similarly, about 50% ATP were derived from respiratory processes in parental MCF7 cells, whereas about 20% ATP were attributed to mitochondrial respiration in MCF7/SIPA1 cells (Figure 2B, right panel). These results strongly suggested that SIPA1 switched energy sources from respiration to fermentation.

We further examined the effect of SIPA1 on the mitochondrial ATP production. When cells were cultured with sodium pyruvate as a sole carbon source, the level of ATP production was significantly higher in SIPA1 low-expression cells than in SIPA1 high-expression cells (Figures 2C,D, green bars). It is, therefore, likely that a high level of SIPA1 expression down-modulated the intracellular ATP generation by switching the ATP source to aerobic glycolysis (Figures 2C,D, right panel). These results suggest that SIPA1 suppressed oxidative phosphorylation in mitochondria of breast cancer cells.

To address whether the alteration of ATP source by SIPA1 affects the sensitivity of cells to glycolytic and mitochondrial inhibitors, we treated the SIPA1-overexpressing or -knockdown breast cancer cells with 10 mM oxamate (an LDH inhibitor) or 50 nM oligomycin. As shown in Figure 2E, suppression of SIPA1 significantly increased the proliferation of MDA-MB-231. When MDA-MB-231 cells were treated with oxamate, the proliferation was significantly decreased, whereas the proliferation of MDA-MB-231 was not affected by the addition of oligomycin. Conversely, treatment of 231si cells with oligomycin greatly suppressed the cell proliferation, while oxamate had no effect on the proliferation of 231si cells. Additionally, overexpressing SIPA1 in MCF7 cells significantly reduced the cell proliferation. Interestingly, oligomycin treatment greatly decreased the proliferation of parental MCF7 cells, while no effect was observed for oxamate treatment. On the contrary, oxamate significantly suppressed the proliferation of MCF7/SIPA1 cells, but oligomycin failed to exhibit the suppressive activity (Figure 2F).

Pyruvate dehydrogenase kinase 1 (PDK1) is a key switch of tricarboxylic acid (TCA) cycle in mitochondria and inhibits mitochondrial oxidation of glucose by blocking the catalytic activity of pyruvate dehydrogenase (PDH) that converts pyruvate to acetyl-CoA (Dupuy et al., 2015). Interestingly, knocking down SIPA1 down-regulated PDK1 at both mRNA and protein expression levels in MDA-MB-231 and BT549 cells, whereas overexpression of SIPA1 up-regulated PDK1 expression in 231si and MCF7 cells (Figures 2G,H and Supplementary Figure S2). It is thus likely that a high level of SIPA1 expression alters the glucose metabolism from respiration to aerobic glycolysis by promoting the expression of glycolysis-related gene products and inhibiting the mitochondrial TCA cycle *via* up-regulating PDK1.

### SIPA1 Promotes *EPAS1* Transcription by Binding to its Promoter

**FIGURE 3:**
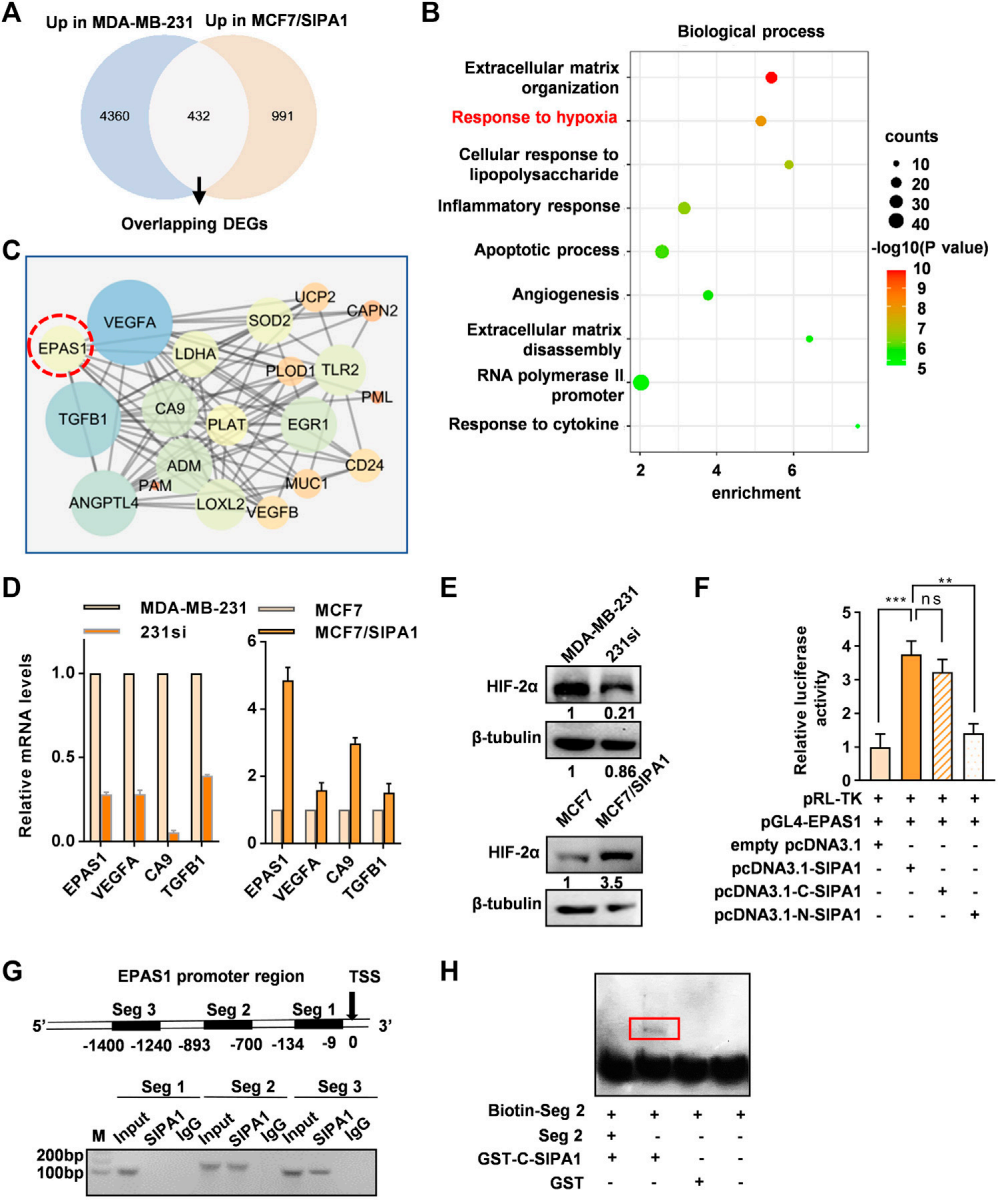
SIPA1 upregulates EPAS1 transcription by binding to its promoter region. **(A)** Transcriptome sequencing datasets of two pairs of cells (MDA-MB-231 vs 231si, MCF7 vs MCF7/SIPA1) were analyzed and 432 overlapping candidate genes which were positively regulated by SIPA1 were selected in the Venn diagram. **(B)** GO biological process enrichment analyses of the 432 genes positively correlated with SIPA1. Top nine GO terms were listed with *p* values, counts and enrichment scores. **(C)** Genes allocated to “responses to hypoxia” in **(B)** were plotted as a protein-protein interaction network. **(D)** qRT-PCR analyses of EPAS1 and its downstream gene candidates, *TGFB1*, *VEGFA* and *CA9*. Data were shown as mean ± s.d. The experiments were conducted in triplicate. SDHC was included as an endogenous control. **(E)** Western blotting analyses of HIF-2α expression in breast cancer cells with various SIPA1 levels. β-tubulin was included as an internal control. **(F)** The effect of SIPA1 on *EPAS1* promoter activity were accessed by a luciferase reporter assay. **(G)** Interaction of SIPA1 with *EPAS1* gene promoter revealed by ChIP-PCR. Seg 1, Seg 2 and Seg 3 represented the indicated promoter regions. TSS, transcription start site. **(H)** Interactions of truncated SIPA1 protein (540-1042aa) with *EPAS1* gene promoter segment 2 were revealed by EMSA. Data shown are mean ± s.d. of triplicate measurements (n = 3).

In order to elucidate the pathway by which SIPA1 modulates the expression of glycolysis-related genes and *PDK1*, we analyzed RNA-seq data from two pairs of breast cancer cell lines: parental and SIPA1-knowndown MDA-MB-231 cells and parental and SIPA1-overexpressing MCF7 cells. Differentially-expressed genes (DEGs) analysis revealed that 4,972 genes were down-regulated and 5,726 genes were up-regulated by knocking-down SIPA1 in MDA-MB-231 cells. In case of overexpressing SIPA1 in MCF7 cells, 1,423 genes were up-regulated and 1,352 genes were down-regulated. The DEGs which were positively correlated with SIPA1 expression in both cell lines were selected, from which 432 bilateral candidate genes were extracted (Figure 3A). Then 432 genes were subjected to GO enrichment analysis, and two annotations named “extracellular matrix organization” and “response to hypoxia” with significant enrichment score and statistically significant *p*-values were noted among the top 9 annotations (Figure 3B). We plotted the protein-protein interaction (PPI) network of the DEGs nominated in the annotation of “response to hypoxia” involved in cellular hypoxic responses as well as aerobic glycolysis processes. *EPAS1* that encodes HIF-2α was identified as a hub gene to link its downstream genes including *TGFB1*, *VEGFA* and *CA9* in sight of the PPI network (Figure 3C). The mRNA levels of *EPAS1* as well as *TGFB1*, *VEGFA* and *CA9*, were confirmed to be positively correlated with those of SIPA1 in these two pairs of breast cancer cells (Figure 3D) and BT549, another TNBC cell line (Supplementary Figure S3). Meanwhile, knocking-down SIPA1 decreased HIF-2α expression in MDA-MB-231 cells, and overexpressing SIPA1 in MCF7 cells increased HIF-2α expression at a protein level (Figure 3E).

It was previously demonstrated that SIPA1 up-regulated the promoter activity of certain target genes in breast cancer cells (Zhang et al., 2015; Wang et al., 2020). In this study, we also demonstrated that SIPA1 could be located in the nuclei of MCF7/SIPA1 and three TNBC cells (Supplementary Figure S4). Dual luciferase reporter assay further revealed that overexpression of SIPA1 and its C-terminal region (540-1042aa), but not the N-terminal region (1-539aa), increased the transcriptional activity of the *EPAS1* promoter transcriptional activity by three-fold, compared to controls (Figure 3F). We then conducted a chromatin immunoprecipitation (ChIP) assay to examine the direct interaction between SIPA1 and the *EPAS1* promoter. Three pairs of specific primers corresponding to three segments of the *EPAS1* promoter, Seg 1, Seg 2, and Seg 3 were employed (Figure 3G, upper panel). As shown in the lower panel of Figure 3G, Seg 2 (−1,400 to −1,240 bp) and Seg 3 (−893 to −700 bp) were specifically amplified from the cell lysates immunopecipitatated with anti-SIPA1 antibody. Moreover, the direct interaction between SIPA1 protein and the *EPAS1* promoter segments was observed by EMSA, in which GST-tagged C-terminal SIPA1 specifically pulled down the biotin-labeled Seg 2, (Figure 3H). It is thus likely that SIPA1 directly interacted with the *EPAS1* promoter, enhanced its promoter activity and promoted HIF-2α expression in breast cancer cells.

### Knocking Down HIF-2α Suppresses Aerobic Glycolysis and invasion of Breast Cancer Cells

**FIGURE 4:**
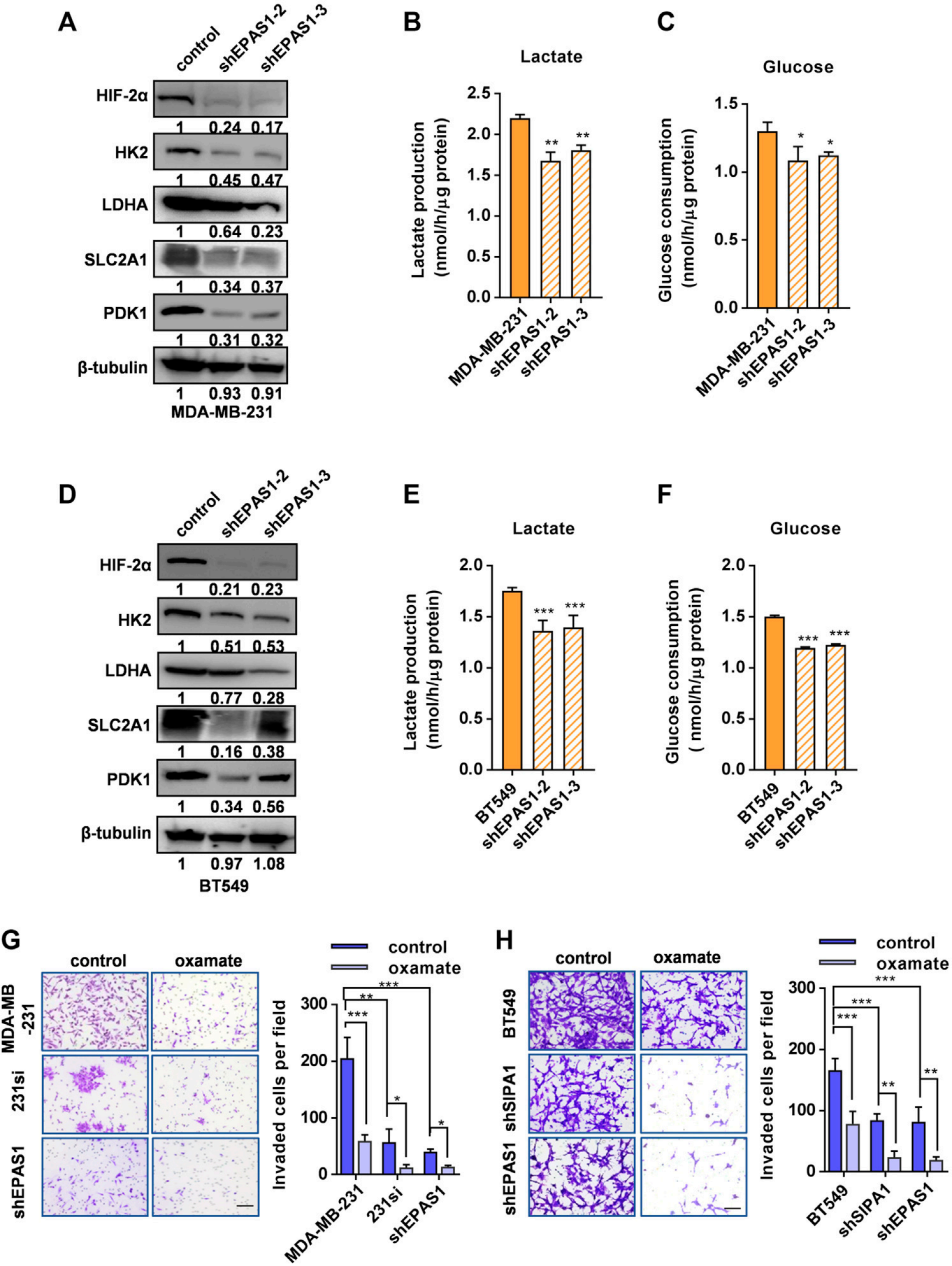
Knocking down HIF-2α suppresses aerobic glycolysis and invasion of breast cancer cells. **(A)** HIF-2α and glycolytic-related proteins in parental and HIF-2α-knockdown MDA-MB-231 cells were determined by Western blotting analysis. β-tubulin was included as an internal control. Production of lactate **(B)** and glucose consumption **(C)** in parental and HIF-2α knockdown MDB-MB-231 cells. **(D)** HIF-2α and glycolysis-related protein levels were determined by Western blotting in parental and HIF-2α knockdown BT549 cells. β-tubulin was included as an internal control. Production of lactate **(E)** and glucose consumption **(F)** in parental and HIF-2α knockdown BT549 cells. **(G–H)** Invasion of MDA-MB-231 **(G)** and BT549 **(H)** and their derivatives treated with or without 20 mM oxamate was analyzed by a transwell assay sistern. Data are shown as mean ± s.d of triplicate measurements (n = 3). *p < 0.05, **p < 0.01, ***p < 0.001. (Student^’^s *t*-test).

We next established stable HIF-2α knockdown MDA-MB-231 cell lines (Figure 4A) to address the hypothesis that the increased dependence of SIPA1-overexpressing breast cancer cells on aerobic glycolysis was mediated by HIF-2α. It was demonstrated that knocking-down HIF-2α in MDA-MB-231 breast cancer cells significantly decreased the expression of glycolysis-related gene products, such as HK2, LDHA, SLC2A1 and PDK1 (Figure 4A), consisting with the suppression of mRNA levels of glycolysis- and hypoxia-related genes (Supplementary Figure S5). In addition, lactate production and glucose consumption were also decreased in MDA-MB-231 cells by knocking down of HIF-2α (Figures 4B,C). Essentially, the same results were observed in BT549 breast cancer cells (Figures 4D–F).

We then compared the invasiveness of the breast cancer cells with SIPA1 or HIF-2α knockdown with that of parental cells by using a transwell assay. As shown in Figure 4G, knocking down either SIPA1 or HIF-2α significantly inhibited the invasion of MDA-MB-231 cells *in vitro* and the tumor cell translocation was further inhibited by the treatment with 20 mM oxamate. BT549 breast cancer cells behaved similarly after gene manipulation and drug treatment (Figure 4H). These results suggest that SIPA1 could enhance the aerobic glycolysis by up-regulating the expression of HIF-2α to promote the invasion of breast cancer cells *in vitro*.

### Blockade of SIPA1/HIF-2α-Mediated Aerobic Glycolysis Suppresses Breast Cancer Growth and Metastasis in Xenografted Mice

**FIGURE 5:**
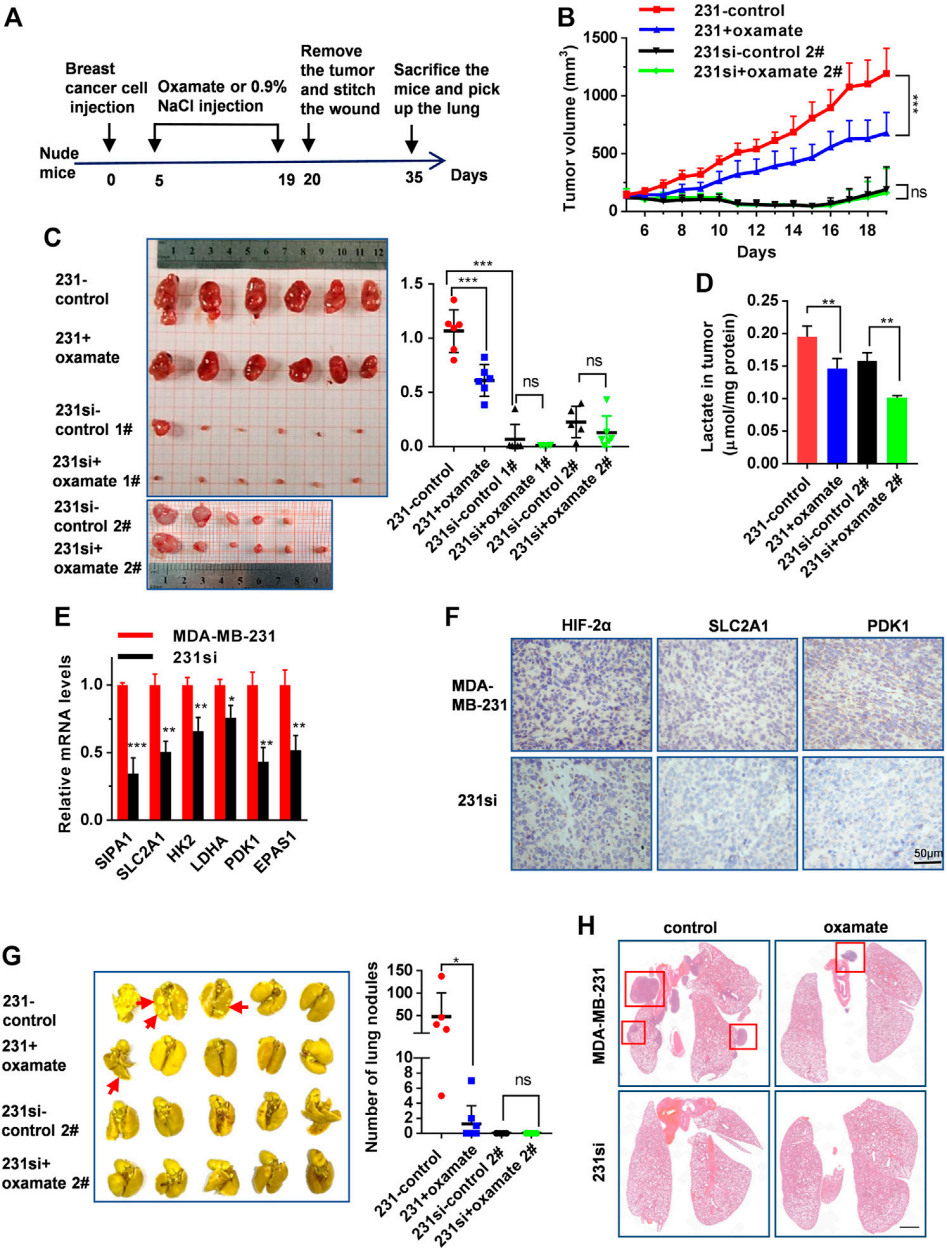
SIPA1/HIF-2α axis blockade suppresses aerobic glycolysis and breast cancer growth and metastasis in a xenograft mouse model. **(A)** The schematic overview of the xenograft experimental workflow. Oxamate treatment on 231si cell xenografted tumor formation experiment was done twice (1# and 2#). **(B)** Tumor volumes in the four groups of mice were measured from day 5 to day 19. **(C)** The tumors in each group were photographed and weighed. **(D)** Lactate levels in tumor tissues were determined in each group, n = 3. **(E)** mRNA levels of glycolytic rate-limiting genes in tumor tissues of mice (n = 3 in each group) were determined by qRT-PCR. Data are shown as mean ± *s*.d. The experiments were done in triplicate. **(F)** Immunohistochemical analyses of HIF-2α, SLC2A1 and PDK1 in tumor tissues derived from two groups of mice were performed. **(G)** Lungs from four groups of mice were photographed (left panel) and the number of nodules observed on the lung surface were counted (right panel). **(H)** Representative histopathological images of HE staining in the lungs were presented. Scale bar: 1 mm. Data are mean ± s.d. of triplicate measurements. *p < 0.05, **p < 0.01, ***p < 0.001. (Student^’^s *t*-test).

We then examined the role of SIPA1/HIF-2α-mediated aerobic glycolysis in breast tumor growth and metastasis *in vivo*. We set up four groups of mice xenografted with (1) MDA-MB-231 breast cancer cells followed by 0.9% NaCl treatment (n = 6), (2) MDA-MB-231 followed by oxamate treatment (n = 6), (3) 231si cells followed by 0.9% NaCl treatment (1#: n = 6, 2#: n = 6), and (4) 231si cells followed by oxamate treatment (1#: n = 6, 2#: n = 6) under the xenograft experimental workflow (Figure 5A). No significant difference was observed on the body weight of mice in all the four groups during the treatments (Supplementary Figure S6). For two groups of mice inoculated with MDA-MB-231 cells, oxamate treatment significantly suppressed the tumor volumes and weights, compared to the group without oxamate treatment. As for the mice inoculated with 231si cells, the tumor growth rate was significantly lower than that of mice xenografted with parental MDA-MB-231 cells (Figures 5B,C).

Furthermore, the level of lactate in MDA-MB-231 xenograft tumors was much higher than that in 231si xenograft tumors. On the other hand, oxamate treatment decreased the level of lactate both in MDA-MB-231 and 231si xenograft tumors (Figure 5D). Also, SIPA1 knockdown decreased the expressions of EPAS1, glycolysis-related products and PDK1 in tumor tissues at mRNA and protein levels (Figures 5E,F).

Regarding lung metastasis, the number of nodules on the surface of the lungs in 231si xenografted mice was significantly fewer than that in MDA-MB-231 xenografted mice. In addition, the oxamate treatment further reduced the number of lung nodules in MDA-MB-231 xenografted mice (Figure 5G). The histopathological images of HE staining revealed that the numbers of tumors and the area of each nodule were decreased in the lungs in mice received 231si cells, compared to those inoculated with MDA-MB-231 cells. In addition, the oxamate treatment reduced the number of tumor nodules (Figure 5H). These results indicate that knocking down SIPA1 decreased breast tumor metastasis, which might be associated with the suppression of glycolysis.

### HIF-2α expression Positively Correlates With SIPA1 Expression and Poor Clinical Outcomes

**FIGURE 6:**
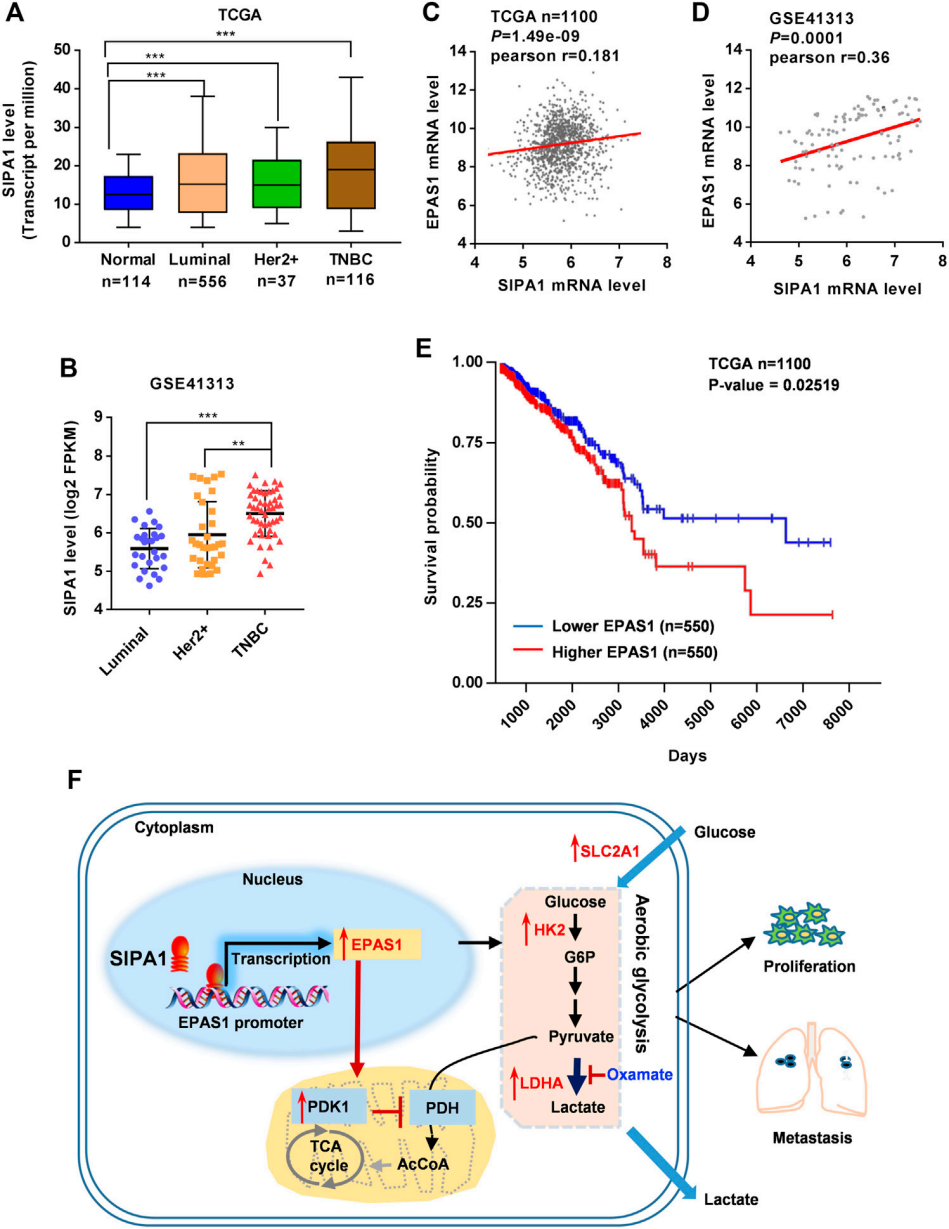
SIPA1/EPAS1 axis is a poor prognosis predictor of breast cancer. **(A–B)** mRNA level of SIPA1 in breast cancer subtypes in TCGA **(A)** and GSE41313 dataset **(B)**. **(C–D)** The relationship between SIPA1 and EPAS1 was analyzed based on TCGA **(C)** and GSE41313 datasets **(D)**. Pearson correlation coefficients (Pr) for SIPA1 and EPAS1 gene expression levels were shown. **(E)** Kaplan-Meier survival analysis for survival probability of breast cancer patient in TCGA according to EPAS1 expression status. The *p* value was presented using the log-rank test. **(F)** A proposed diagram for SIPA1-mediated aerobic glycolysis and breast cancer cell metastasis.

To determine the clinical relevance of the above findings in breast cancers, we analyzed the TCGA database and found that SIPA1 was up-regulated in some breast cancer subtypes including luminal, Her2+ and TNBC (Figure 6A). In addition, we found that SIPA1 was aberrantly expressed in TNBC cells when compared to the other subtypes in GSE41313 dataset (Figure 6B). We also found a positive correlation between mRNA levels of *SIPA1* and *EPAS1* genes in both datasets (Figures 6C,D). A survival analysis by the log-rank test indicated that patients with *EPAS1*-high tumors had significantly lower rates of survival probability than patients with *EPAS1*-low tumors in TCGA (Figure 6E). These data suggest that SIPA1 induced the expression of HIF-2α and the expression of HIF-2α in tumor cells might result in poor prognosis in breast cancer patients.

Taken together, SIPA1/HIF-2α axis could be a key regulator of aerobic glycolysis, contributing to the switch from oxidative phosphorylation to aerobic glycolysis in breast cancer cells under an ambient oxygen condition, leading to tumor invasion and metastasis (Figure 6F).

## Discussion

Accumulating evidence indicates that SIPA1 is highly expressed in various solid tumors and that its expression is correlated with metastasis and poor prognosis in many types of cancers (Liu et al., 2020). In the present study, we demonstrated that SIPA1 was aberrantly expressed in some breast cancer cells, especially in TNBC cells, and promoted aerobic glycolysis, leading to tumor invasion and metastasis *in vivo*.

It has been reported that HIF-1α and c-myc are two master regulators of aerobic glycolysis by targeting multiple glycolysis-related genes. Under normoxic conditions, however, SIPA1 enhanced aerobic glycolysis without affecting the expression of HIF-1α and c-myc in breast cancer cells, suggesting additional mechanisms rather than HIF-1α/c-myc pathway are involved.

SIPA1 knockdown led to down-regulation of nearly all glycolysis-related genes and increased dependence on respiration, instead of glycolysis, in the generation of ATP, suggesting that the network for glycolytic flux in breast cancer cells could be modulated by SIPA1. It is noted that SIPA1 decreased a total cellular ATP content in spite of marked enhancement of aerobic glycolysis. In fact, SIPA1 shifted ATP production from mitochondrial respiration to fermentation *via* aerobic glycolysis. It has been reported that an increase in glycolysis, especially aerobic glycolysis, and a decrease in mitochondrial functions are characteristic of breast cancer cells with a metastatic phenotype (Lu et al., 2010). On the contrary, other reports indicated that an increase in mitochondrial activity was also important for the metastatic phenotype (LeBleu et al., 2014; Dupuy et al., 2015; Andrzejewski et al., 2017). In the present study, we showed that metabolic transformation to decreased glycolysis and increased mitochondrial oxidative phosphorylation induced by SIPA1 knockdown led to poor metastatic properties of breast cancer cells. It suggests that breast cancer cells exhibit metabolic plasticity that balances energy production sources between glycolysis and mitochondrial oxidative phosphorylation and modulates proliferation and metastatic properties.

In the present study, we comprehensively searched for candidate genes that might link between SIPA1 and glycolysis-related factors by employing transcriptome analyses. We then demonstrated that HIF-2α (encoded by *EPAS1*) was directly regulated by SIPA1 in breast cancer cells. It was previously shown that HIF-2α protein was gradually accumulated in tumor cells and contributed to the prolonged activation of hypoxia-related genes, in which HIF-2α regulated a glycolytic flux by targeting several glycolysis-related genes such as SLC2A1 and LDHA. In addition, HIF-2α was shown to regulate angiogenesis-related signaling pathways by targeting genes including vascular endothelial growth factor A (VEGFA) to promote an aggressive phenotype of tumor cells (Holmquist-Mengelbier et al., 2006; Chen et al., 2017).

Herein, we found that SIPA1 could enhance HIF-2α expression and that HIF-2α knockdown decreased the aerobic glycolytic flux and expression of glycolysis-related genes such as HK2, SLC2A1, and LDHA under normoxia, suggesting that HIF-2α is a potent mediator controlling the hypoxic phenotype including aerobic glycolysis. Under normoxia, HIF-1α protein could be hydroxylated on the proline residues due to the action of prolyl hydroxylase 2 and be rapidly digested by 26S proteasomes. (Semenza, 2003). HIF-2α protein is, however, more stable and active than HIF-1α under resembling end capillary oxygen conditions (Holmquist-Mengelbier et al., 2006). Whereas it is challenging to address the long-standing mystery why cancer cells adapt the less efficient glycolysis for energy sources even with ambient oxygen supply, it would be important to shed light on the SIPA1/HIF-2α axis that regulates aerobic glycolysis under normoxia for better understanding of cancer metastasis.

Interestingly, 231si cells with reduced expression of SIPA1 grew faster than the parental MDA-MB-231 cells at 48 h under a normoxic condition *in vitro*. In contrast, the xenografted tumor in the mice inoculated with MDA-MB-231 cells was much larger than that in mice injected with 231si cells, indicating that MDA-MB-231 cells grew faster than 231si cells *in vivo*. In our study, MDA-MB-231 cells were characterized by a glycolytic phenotype evidenced by higher glucose consumption, higher lactate production, higher expression of glycolytic genes, main source of glycolytic ATP, and lower mitochondrial activity due to high expression of PDK1 (a mitochondrial inhibitor, which inhibits conversion of pyruvate into acetyl-CoA). Given that 231si cells were equipped with functionally active mitochondria to generate much more ATP than MDA-MB-231 cells, evidenced by their lower expression of glycolytic genes and PDK1, the mitochondria provide advantages for 231si cells in proliferation under normoxia *in vitro*. Tumor cells generally undergo hypoxia condition *in vivo* due to limited supply and increased demand of oxygen during rapid expansion (Hanahan and Weinberg, 2011). This context preferentially supports MDA-MB-231 cells favoring glycolytic metabolism.

In the present study, we demonstrated that SIPA1 could up-regulate the expression of PDK1 through HIF-2α. PDK1 was originally characterized as a suppressor of TCA cycle by inhibiting PDH activity to decrease the conversion of pyruvate to acetyl-CoA in mitochondria. Breast cancer cells highly expressing SIPA1 greatly depend on glycolysis in the production of ATP, and thus have advantages in cell proliferation under hypoxia like in solid tumor tissues. In fact, SIPA1 knockdown breast cancer cells exhibited impaired growth in xenografted mice, whereas rapid proliferation was observed *in vitro* under normoxia.

Animal models further showed that the SIPA1/HIF-2α axis could play an important role in aerobic glycolysis and metastasis of breast cancer cells *in vivo*. SIPA1 knockdown as well as LDH inhibitor oxamate administration markedly inhibited breast cancer cell metastasis from primary xenografted sites to the lungs, suggesting that the blockade of glycolysis mediated by SIPA1/HIF-2α axis is an efficacious strategy for the treatment of aggressive breast cancer that expresses SIPA1. Taken together, the present study revealed a novel regulatory mechanism how breast cancer cells, especially TNBC cells highly expressing SIPA1, facilitated cancer progression *via* a metabolic shift from respiration to aerobic glycolysis.

## Materials and Methods

### Plasmids and Cells

Plasmids of pcDNA3-SIPA1, pcDNA3-N-SIPA1 encoding N-terminal half part of SIPA1 (1-539aa) and pcDNA3-C-SIPA1 encoding C-terminal half part of SIPA1 (540-1042aa) were constructed as previously described (Ma et al., 2021). SIPA1 and EPAS1 shRNA nucleotides (Supplementary Table S1) were cloned into pLKO.1-GFP (RRID: Addgene_30323) to construct pLKO.1-GFP-SIPA1 and pLKO.1-GFP-EPAS1, respectively. Lentivirus packaging plasmids, psPAX2 (RRID: Addgene_12260) and pMD2. G (RRID: Addgene_12259), and a reporter plasmid pRL-TK (RRID: Addgene_11313) were purchased from Merck & Co. (Kenilworth, NJ). EPAS1 promoter region (-1618bp to transcription starting site) was cloned into pGL4 (RRID: Addgene_48744) to give pGL4-EPAS1. pGEX-C-SIPA1 was constructed to express GST-tagged C-terminal half part SIPA1 protein.

Human cell lines MDA-MB-231, MCF7, and HEK293T were purchased and authorized from China Center for Type Culture Collection (CCTCC, Wuhan, China). BT549 was purchased from Procell (Wuhan, China) with short tandem repeat authentication. SUM159 were gifted from Dr. Peijing Zhang (Yao et al., 2018). Cells were cultured according to the previous study (Ma et al., 2021). The stable knockdown cells were established following the previous study (Zhang et al., 2015).

### Measurement of Glucose Consumption, Lactate Production, Intracellular and Extracellular Lactate Dehydrogenase Activity

To detect glucose consumption and lactate production, 5 × 10^4^ cells were placed into wells and incubated in modified RPMI1640 medium with 15 mM glucose in the absence of glutamine and pyruvate for 5 h. After being washed with PBS, the culture medium and cells were harvested separately. Concentrations of glucose and lactate in the culture medium were measured using a glucose assay kit and a lactate assay kit (Nanjing Jiancheng Bioengineering Institute, Nanjing, China) according to the manufacturer’s instructions, respectively. To measure lactate in tumor tissues, 30 mg tumor specimens were pulverized in liquid nitrogen and lysed in 300 µl NP40 lysis buffer. After centrifugation for 5 min at 13,000 rpm at 4oC, the supernatant was harvested and examined for lactate and protein concentrations.

For the determination of the intracellular lactate dehydrogenase (LDH) activity, 1 × 10^5^ cells were seeded into the wells and incubated in media for 24 h. After being washed with PBS, cells were harvested and examined for LDH activity with an LDH assay kit (Nanjing Jiancheng Bioengineering Institute) according to the manufacturer’s instructions. For extracellular LDH activity assay, 2 ×10^5^ cells were seeded into the wells and incubated with medium for 5 h, and then supernatants were collected and examined for LDH activity by using an LDH assay kit.

### Measurement of ATP Level

ATP levels in breast cancer cells were measured using an ATP assay kit (Beyotime, Nanjing, China) according to the manufacturer’s instructions. The amount of total ATP was calculated based on luminescence measured on a FlexStation 3 luminescence reader (Molecular Devices, San Jose, CA). To measure the levels of glycolytic ATP production, 5 × 10^4^ cells were seeded into wells and incubated in media containing 100 nM oligomycin (Thermo Fisher Scientific) for 5 h. After being washed with PBS, the cells were lysed and examined for ATP levels using an ATP assay kit according to the manufacturer’s protocol. To measure the ATP production in mitochondria, 5 × 10^4^ cells were seeded into wells and incubated in media containing 10 mM pyruvate in the absence of glucose and glutamine for 5 h. After being washed with PBS, the cells were lysed and examined for ATP levels and protein concentrations.

### qRT-PCR and ChIP-PCR

Quantitative real-time polymerase chain reaction (qRT-PCR) and chromatin immunoprecipitation PCR (ChIP-PCR) were conducted as previously described (Zhang et al., 2015). The specific primers are listed in Supplementary Tables S2 and S3.

### Transcriptome Sequencing and Analysis

Total RNA was isolated using TRIzol reagent (Thermo Fisher Scientific). Transcriptome sequencing was conducted by Novogene Co. Ltd. (Beijing, China). Gene expression levels for each transcript were estimated as the number of reads per kilobase of exon model per million mapped reads (RPKM) using only uniquely mapped reads in exonic regions. A gene was considered differentially expressed if its expression differed between any two samples with the fold change >2 and the *p* value <0.05. DAVID (RRID: SCR_001881) online tool (https://david.abcc.ncifcrf.gov) was used for Gene Ontology (GO) enrichment analysis and KEGG (RRID: SCR_012773) pathway enrichment analysis. STRING (RRID: SCR_005223) online tool (https://string-db.org) was used to assess the protein-protein interaction of cluster genes.

### Luciferase Reporter Assay

EPAS1 promoter region was amplified by PCR from MDA-MB-231 genomic DNA, and cloned into a pGL-4 basic luciferase expression vector (Promega, Madison, WI). Reporter assays were performed using HEK293T cells transfected with the indicated plasmids and analyzed using a Dual-Luciferase Reporter Assay kit (Promega). The luciferase activity was measured by FlexStation 3 (Molecular Devices). The expression levels were normalized with respect to those for cells co-transfected with a renilla plasmid.

### Western Blotting and Immunohistochemistry

Cell lyzates, cytoplasmic lyzates, and nuclear extracts were prepared as previously described (Zhang et al., 2015). Primary antibodies for SIPA1 (Abcam, ab85928, RRID: AB_1925436), LDHA (Abcam, ab47010, RRID: AB_1952042), HK2 (Abcam, ab104836, RRID: AB_10710018), SLC2A1 (Abcam, ab115730), HIF-2α (Abcam, ab207607, RRID: AB_2618694), HIF-1α (Abcam, ab179483), c-myc (Cell Signaling Technology, Danvers, MA, 9,402), PDK1 (Abcam, ab207450), and secondary antibodies (Cell Signaling Technology, 91196 and 7,074) were used for blotting, and β-tubulin (Absin Bioscience Inc., Shanghai, China, abs830032) was included as a control. The relative amount of protein was quantitated by an ImageJ software.

For immunohistochemistry, the xenograft tumor tissue slides underwent deparaffinization, rehydration, and antigen-retrieval and were then incubated with the primary antibody: HIF-2α (Abcam, ab207607), SLC2A1 (Abcam, ab115730) or PDK1 (Abcam, ab207450). Staining image was monitored using 80i fluorescence microscope (Nikon, Tokyo, Japan).

### Electrophoretic Mobility Shift Assay

GST-tagged C-terminal half of SIPA1 was expressed in *E. coli* BL21 (DE3) and purified using glutathione-Sepharose beads. The purified protein (2 µg) was incubated with competitive biotin-labeled double-stranded DNA segments on ice for 5 min. Then, 1 µ mole of unlabeled double-stranded DNA segments, which had been amplified from EPAS1 promoter, were added to the mixture, and the protein/DNA mixture was incubated at room temperature for 30 min. The proteins bound to biotin-labeled Seg 2 were resolved by native 6.5% PAGE and imaged by immunoblot assay.

### Cell Proliferation and invasion Assay

Cell growth and invasion *in vitro* was measured as described previously (Zhang et al., 2015).

### Animal Studies

All animal studies were conducted after approval by the Institutional Animal Care Committee of Huazhong University of Science and Technology. For xenograft experiments, female BALB/c nude mice were divided into two groups, and MDA-MB-231 and 231si cells (2 × 10^6^/mouse) were subcutaneously injected with matrigel (v/v = 3:1) into the right mammary pad for each group, respectively. Five days later, a subset of 6 mice in each group received an intraperitoneal injection with 15 mg oxamate or 0.9% NaCl. The administration was done every 24 h for the next 14 days. The tumor volume was determined every 24 h with vernier caliper according to the following formula: volume (mm^3^) = [width (mm)]^2^ × [length (mm)]/2. Subsequently, all the tumors were surgically removed and weighed, and the wounds were stitched with surgical suture to maintain animals for 15 additional days. Finally, all the mice were sacrificed by cervical dislocation, and the lungs of each mouse were dissected and immersed in formalin, then analyzed by haematoxylin and eosin (HE) staining.

### GEO and TCGA Data Analysis

Dataset GSE41313 was downloaded from GEO (Riaz et al., 2013). In GSE41313, mRNA expression levels in 153 samples from 51 breast cancer cell lines were extracted from the general public license (GPL) 13158 platform (Affymetrix, Santa Clara, CA). Fragments per kilobase of exon model per million mapped fragments (FPKM) were used to compare gene expressions in different groups. Data acquisition and application from The Cancer Genome Atlas (TCGA) were performed in accordance with TCGA publication guidelines and data access policies (Chandrashekar et al., 2017).

### Statistical Analysis

The data were presented as mean ± s.d. Student’s t-test was used to evaluate *p* values. One-way or two-way ANOVA were used to compare multiple testing correction within multiple groups. A *p*-value of 0.05 or lower is considered to be statistically significant.

### Associated Data

The raw sequence data reported in this paper have been deposited in the Genome Sequence Archive (Chen et al., 2021) in National Genomics Data Center (CNCB-NGDC Members and Partners, 2021), China National Center for Bioinformation/Beijing Institute of Genomics, Chinese Academy of Sciences, under accession number HRA001265 that are publicly accessible at https://ngdc.cncb.ac.cn/gsa-human.

## Data Availability

The datasets presented in this study can be found in online repositories. The names of the repository/repositories and accession number(s) can be found in the article/Supplementary Material.

## References

[B1] Andrzejewski S. Klimcakova E. Johnson R. M. Tabariès S. Annis M. G. McGuirk S. (2017). PGC-1α Promotes Breast Cancer Metastasis and Confers Bioenergetic Flexibility against Metabolic Drugs. Cel Metab. 26, 778–787. 10.1016/j.cmet.2017.09.006 28988825

[B2] Chandrashekar D. S. Bashel B. Balasubramanya S. A. H. Creighton C. J. Ponce-Rodriguez I. Chakravarthi B. V. S. K. (2017). UALCAN: A Portal for Facilitating Tumor Subgroup Gene Expression and Survival Analyses. Neoplasia 19, 649–658. 10.1016/j.neo.2017.05.002 28732212 PMC5516091

[B3] Chen D. Wu L. Liu L. Gong Q. Zheng J. Peng C. (2017). Comparison of HIF1A-AS1 and HIF1A-AS2 in Regulating HIF-1α and the Osteogenic Differentiation of PDLCs under Hypoxia. Int. J. Mol. Med. 40, 1529–1536. 10.3892/ijmm.2017.3138 28949371

[B4] Chen T. Chen X. Zhang S. Zhu J. Tang B. Wang A. (2021). The Genome Sequence Archive Family: Toward Explosive Data Growth and Diverse Data Types. Genomics Proteomics Bioinformatics, S1672-0229(21)00163-7. 10.1016/j.gpb.2021.08.001 PMC9039563 34400360

[B5] CNCB-NGDC Members and Partners (2021). Database Resources of the National Genomics Data Center, China National Center for Bioinformation in 2021. Nucleic Acids Res. 49, D18–D28. 10.1093/nar/gkaa1022 36420893 PMC9825504

[B6] Denko N. C. (2008). Hypoxia, HIF1 and Glucose Metabolism in the Solid Tumour. Nat. Rev. Cancer 8, 705–713. 10.1038/nrc2468 19143055

[B7] Dupuy F. Tabariès S. Andrzejewski S. Dong Z. Blagih J. Annis M. G. (2015). PDK1-Dependent Metabolic Reprogramming Dictates Metastatic Potential in Breast Cancer. Cel Metab. 22, 577–589. 10.1016/j.cmet.2015.08.007 26365179

[B8] García-Cañaveras J. C. Chen L. Rabinowitz J. D. (2019). The Tumor Metabolic Microenvironment: Lessons from Lactate. Cancer Res. 79, 3155–3162. 10.1158/0008-5472.can-18-3726 31171526 PMC6606343

[B9] Gatenby R. A. Gillies R. J. (2004). Why Do Cancers Have High Aerobic Glycolysis? Nat. Rev. Cancer 4, 891–899. 10.1038/nrc1478 15516961

[B10] Hanahan D. Weinberg R. A. (2011). Hallmarks of Cancer: the Next Generation. Cell 144, 646–674. 10.1016/j.cell.2011.02.013 21376230

[B11] Holmquist-Mengelbier L. Fredlund E. Löfstedt T. Noguera R. Navarro S. Nilsson H. (2006). Recruitment of HIF-1α and HIF-2α to Common Target Genes Is Differentially Regulated in Neuroblastoma: HIF-2α Promotes an Aggressive Phenotype. Cancer Cell 10, 413–423. 10.1016/j.ccr.2006.08.026 17097563

[B12] Hsieh A. L. Walton Z. E. Altman B. J. Stine Z. E. Dang C. V. (2015). MYC and Metabolism on the Path to Cancer. Semin. Cel Develop. Biol. 43, 11–21. 10.1016/j.semcdb.2015.08.003 PMC4818970 26277543

[B13] Jeon H. M. Kim D. H. Jung W.-H. Koo J. S. (2013). Expression of Cell Metabolism-Related Genes in Different Molecular Subtypes of Triple-Negative Breast Cancer. Tumori 99, 555–564. 10.1177/030089161309900419 24326847

[B14] Ji K. Ye L. Toms A. M. Hargest R. Martin T. A. Ruge F. (2012). Expression of Signal-Induced Proliferation-Associated Gene 1 (SIPA1), a RapGTPase-Activating Protein, Is Increased in Colorectal Cancer and Has Diverse Effects on Functions of Colorectal Cancer Cells. Cancer Genomics Proteomics 9, 321–327. 22990111

[B15] Kawada K. Toda K. Sakai Y. (2017). Targeting Metabolic Reprogramming in KRAS-Driven Cancers. Int. J. Clin. Oncol. 22, 651–659. 10.1007/s10147-017-1156-4 28647837

[B16] Kim J. Yu L. Chen W. Xu Y. Wu M. Todorova D. (2019). Wild-Type P53 Promotes Cancer Metabolic Switch by Inducing PUMA-dependent Suppression of Oxidative Phosphorylation. Cancer Cell 35, 191–203. 10.1016/j.ccell.2018.12.012 30712844

[B17] Kim W. Y. Perera S. Zhou B. Carretero J. Yeh J. J. Heathcote S. A. (2009). HIF2α Cooperates with RAS to Promote Lung Tumorigenesis in Mice. J. Clin. Invest. 119, 2160–2170. 10.1172/jci38443 19662677 PMC2719950

[B18] Kroemer G. Pouyssegur J. (2008). Tumor Cell Metabolism: Cancer's Achilles' Heel. Cancer Cell 13, 472–482. 10.1016/j.ccr.2008.05.005 18538731

[B19] Lanning N. J. Castle J. P. Singh S. J. Leon A. N. Tovar E. A. Sanghera A. (2017). Metabolic Profiling of Triple-Negative Breast Cancer Cells Reveals Metabolic Vulnerabilities. Cancer Metab. 5, 6. 10.1186/s40170-017-0168-x 28852500 PMC5568171

[B20] LeBleu V. S. O’Connell J. T. Gonzalez Herrera K. N. Wikman H. Pantel K. Haigis M. C. (2014). PGC-1α Mediates Mitochondrial Biogenesis and Oxidative Phosphorylation in Cancer Cells to Promote Metastasis. Nat. Cel Biol 16, 992–1003. 10.1038/ncb3039 PMC4369153 25241037

[B21] Liu C. Jiang W. G. Hargest R. Martin T. A. (2020). The Role of SIPA1 in the Development of Cancer and Metastases (Review). Mol. Clin. Oncol. 13, 32. 10.3892/mco.2020.2102 32789016 PMC7416617

[B22] Lu X. Bennet B. Mu E. Rabinowitz J. Kang Y. (2010). Metabolomic Changes Accompanying Transformation and Acquisition of Metastatic Potential in a Syngeneic Mouse Mammary Tumor Model. J. Biol. Chem. 285, 9317–9321. 10.1074/jbc.c110.104448 20139083 PMC2843179

[B23] Ma Y. Weng J. Wang N. Zhang Y. Minato N. Su L. (2021). A Novel Nuclear Localization Region in SIPA1 Determines Protein Nuclear Distribution and Epirubicin-Sensitivity of Breast Cancer Cells. Int. J. Biol. Macromolecules 180, 718–728. 10.1016/j.ijbiomac.2021.03.101 33753200

[B24] Mathieu V. Pirker C. Schmidt W. M. Spiegl-Kreinecker S. Lötsch D. Heffeter P. (2012). Aggressiveness of Human Melanoma Xenograft Models Is Promoted by Aneuploidy-Driven Gene Expression Deregulation. Oncotarget 3, 399–413. 10.18632/oncotarget.473 22535842 PMC3380575

[B25] Pérez-Escuredo J. Dadhich R. K. Dhup S. Cacace A. Van Hée V. F. De Saedeleer C. J. (2016). Lactate Promotes Glutamine Uptake and Metabolism in Oxidative Cancer Cells. Cell Cycle 15, 72–83. 10.1080/15384101.2015.1120930 26636483 PMC4825768

[B26] Rankin E. B. Giaccia A. J. (2016). Hypoxic Control of Metastasis. Science 352, 175–180. 10.1126/science.aaf4405 27124451 PMC4898055

[B27] Riaz M. van Jaarsveld M. T. Hollestelle A. Prager-van der Smissen W. J. Heine A. A. Boersma A. W. (2013). miRNA Expression Profiling of 51 Human Breast Cancer Cell Lines Reveals Subtype and Driver Mutation-specific miRNAs. Breast Cancer Res. 15, R33. 10.1186/bcr3415 23601657 PMC3672661

[B28] Semenza G. L. (2003). Targeting HIF-1 for Cancer Therapy. Nat. Rev. Cancer 3, 721–732. 10.1038/nrc1187 13130303

[B29] Shimizu Y. Hamazaki Y. Hattori M. Doi K. Terada N. Kobayashi T. (2011). SPA-1 Controls the Invasion and Metastasis of Human Prostate Cancer. Cancer Sci. 102, 828–836. 10.1111/j.1349-7006.2011.01876.x 21251160

[B30] Simões R. V. Serganova I. S. Kruchevsky N. Leftin A. Shestov A. A. Thaler H. T. (2015). Metabolic Plasticity of Metastatic Breast Cancer Cells: Adaptation to Changes in the Microenvironment. Neoplasia 17, 671–684. 10.1016/j.neo.2015.08.005 26408259 PMC4674487

[B31] Wada Y. Kubota H. Maeda M. Taniwaki M. Hattori M. Imamura S. (1997). Mitogen-InducibleSIPA1Is Mapped to the Conserved Syntenic Groups of Chromosome 19 in Mouse and Chromosome 11q13.3 Centromeric toBCL1in Human. Genomics 39, 66–73. 10.1006/geno.1996.4464 9027487

[B32] Wang N. Weng J. Xia J. Zhu Y. Chen Q. Hu D. (2020). SIPA1 Enhances SMAD2/3 Expression to Maintain Stem Cell Features in Breast Cancer Cells. Stem Cel Res. 49, 102099. 10.1016/j.scr.2020.102099 33296812

[B33] Wang Z. Jiang Q. Dong C. (2020). Metabolic Reprogramming in Triple-Negative Breast Cancer. Cancer Biol. Med. 17, 44–59. 10.20892/j.issn.2095-3941.2019.0210 32296576 PMC7142847

[B34] Warnecke C. Zaborowska Z. Kurreck J. Erdmann V. A. Frei U. Wiesener M. (2004). Differentiating the Functional Role of Hypoxia‐inducible Factor (HIF)‐1α and HIF‐2α (EPAS‐1) by the Use of RNA Interference: Erythropoietin Is a HIF‐2α Target Gene in Hep3B and Kelly Cells. FASEB j. 18, 1462–1464. 10.1096/fj.04-1640fje 15240563

[B35] Yang H. Geng Y. H. Wang P. Zhou Y. T. Yang H. Huo Y. F. (2019). Extracellular ATP Promotes Breast Cancer Invasion and Epithelial‐mesenchymal Transition via Hypoxia‐inducible Factor 2α Signaling. Cancer Sci. 110, 2456–2470. 10.1111/cas.14086 31148343 PMC6676128

[B36] Yao F. Zhou Z. Kim J. Hang Q. Xiao Z. Ton B. N. (2018). SKP2- and OTUD1-Regulated Non-proteolytic Ubiquitination of YAP Promotes YAP Nuclear Localization and Activity. Nat. Commun. 9, 2269. 10.1038/s41467-018-04620-y 29891922 PMC5995870

[B37] Zhang T. Suo C. Zheng C. Zhang H. (2019). Hypoxia and Metabolism in Metastasis. Adv. Exp. Med. Biol. 1136, 87–95. 10.1007/978-3-030-12734-3_6 31201718

[B38] Zhang Y. Gong Y. Hu D. Zhu P. Wang N. Zhang Q. (2015). Nuclear SIPA1 Activates Integrin β1 Promoter and Promotes Invasion of Breast Cancer Cells. Oncogene 34, 1451–1462. 10.1038/onc.2014.36 24704834

