# Supplementary figures and images for: SIPA1 Enhances Aerobic Glycolysis Through HIF-2α Pathway to Promote Breast Cancer Metastasis
Source: Front Cell Dev Biol. 2022 Jan 12;9:779169. doi: 10.3389/fcell.2021.779169 (PMC8790513; doi:10.3389/fcell.2021.779169)

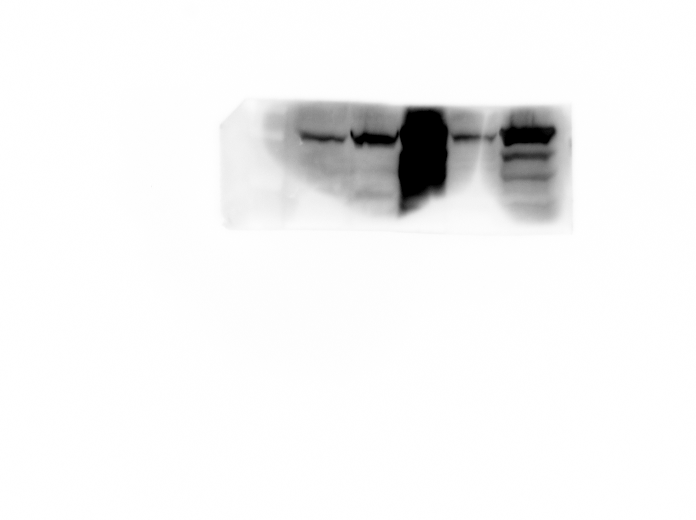

Supplement: Supplementary file 1 [file DataSheet1.ZIP › row images for WB/Figure 1A left SIPA1.tif]

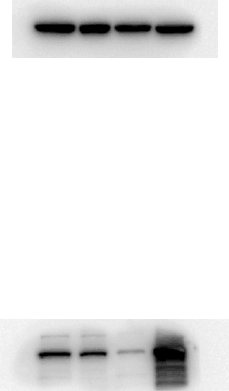

Supplement: Supplementary file 1 [file DataSheet1.ZIP › row images for WB/Figure 1A left tubulin.tif]

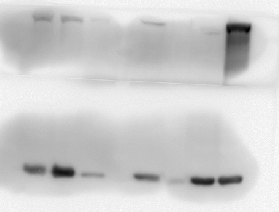

Supplement: Supplementary file 1 [file DataSheet1.ZIP › row images for WB/Figure 1A right SIPA1.tif]

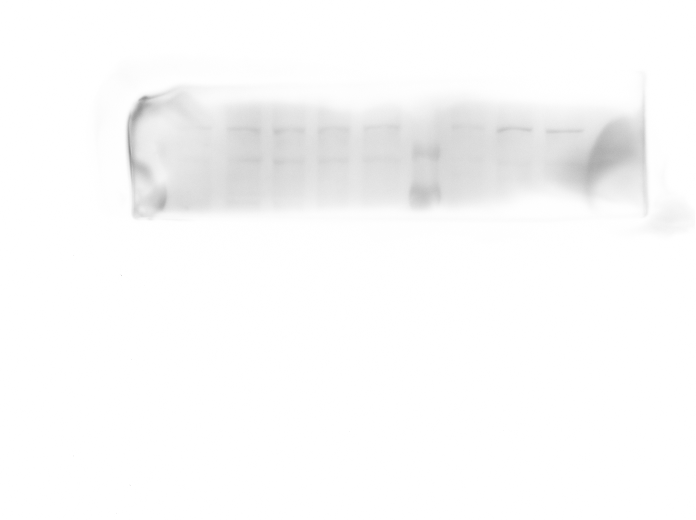

Supplement: Supplementary file 1 [file DataSheet1.ZIP › row images for WB/Figure 1F left HIF1A.tif]

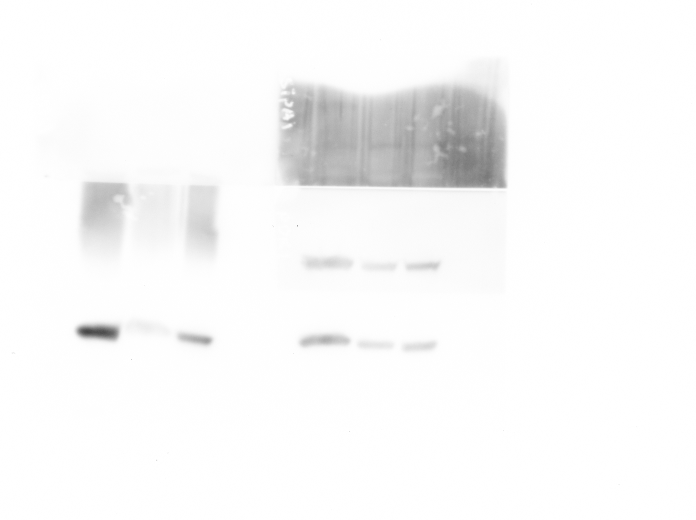

Supplement: Supplementary file 1 [file DataSheet1.ZIP › row images for WB/Figure 1F left HK2.tif]

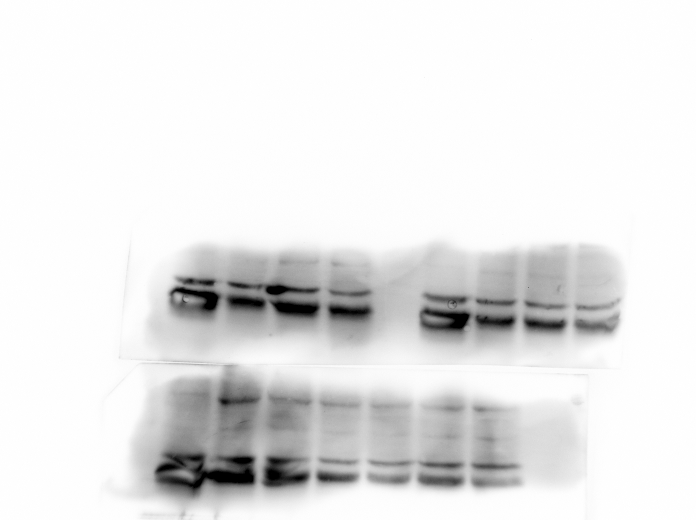

Supplement: Supplementary file 1 [file DataSheet1.ZIP › row images for WB/Figure 1F left LDHA.tif]

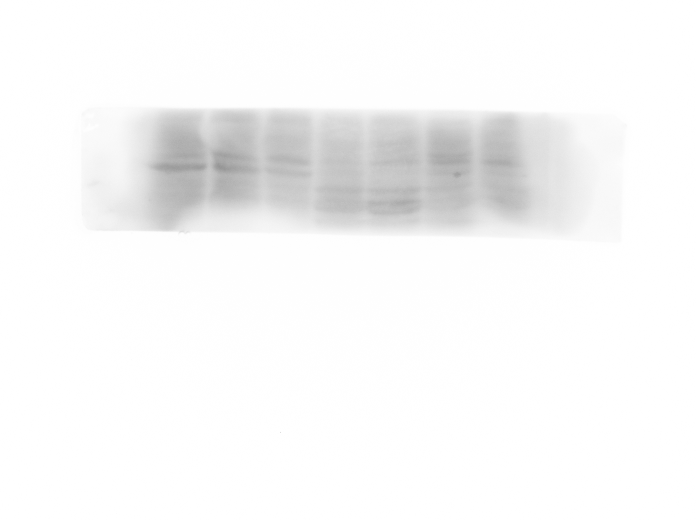

Supplement: Supplementary file 1 [file DataSheet1.ZIP › row images for WB/Figure 1F left c-myc.tif]

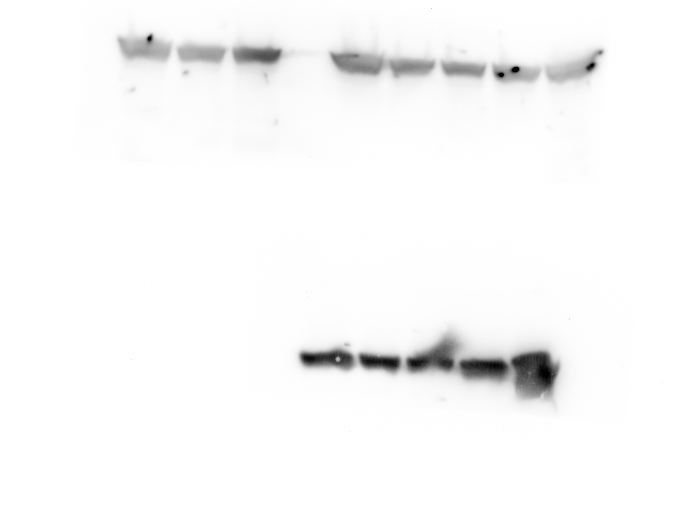

Supplement: Supplementary file 1 [file DataSheet1.ZIP › row images for WB/Figure 1F left tubulin.tif]

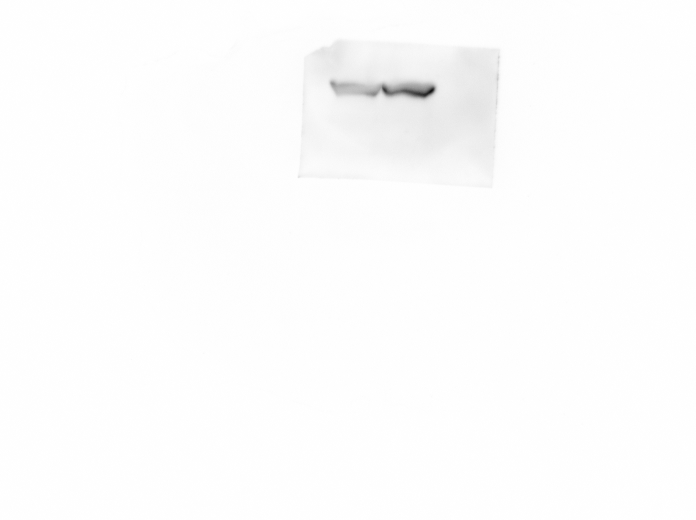

Supplement: Supplementary file 1 [file DataSheet1.ZIP › row images for WB/Figure 1F right HK2.tif]

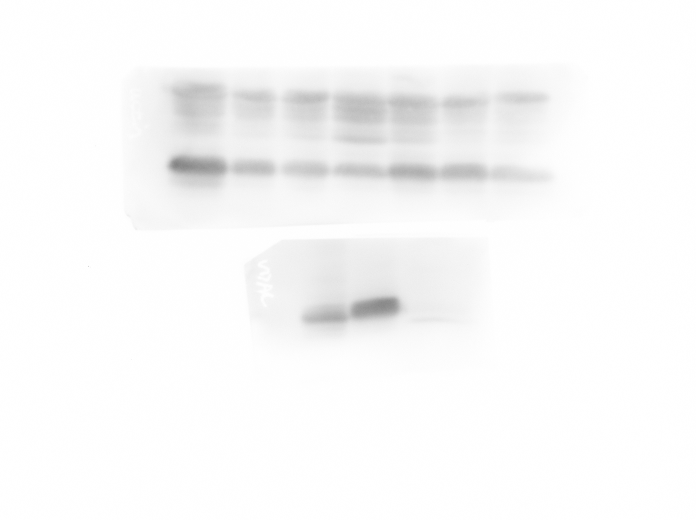

Supplement: Supplementary file 1 [file DataSheet1.ZIP › row images for WB/Figure 1F right LDHA.tif]

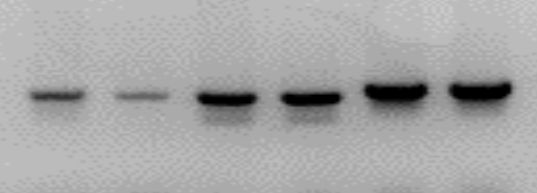

Supplement: Supplementary file 1 [file DataSheet1.ZIP › row images for WB/Figure 1F right tubulin.tif]

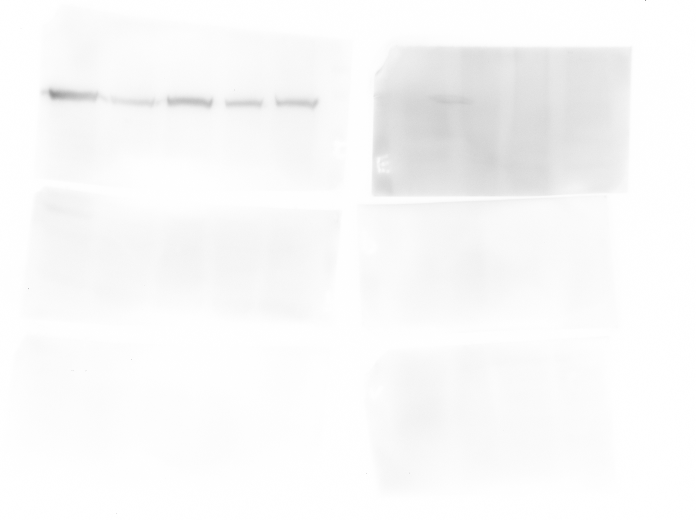

Supplement: Supplementary file 1 [file DataSheet1.ZIP › row images for WB/Figure 2H PDK1 left.tif]

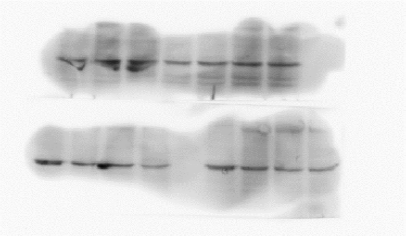

Supplement: Supplementary file 1 [file DataSheet1.ZIP › row images for WB/Figure 2H tubulin left.tif]

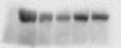

Supplement: Supplementary file 1 [file DataSheet1.ZIP › row images for WB/Figure 2H tubulin right.tif]

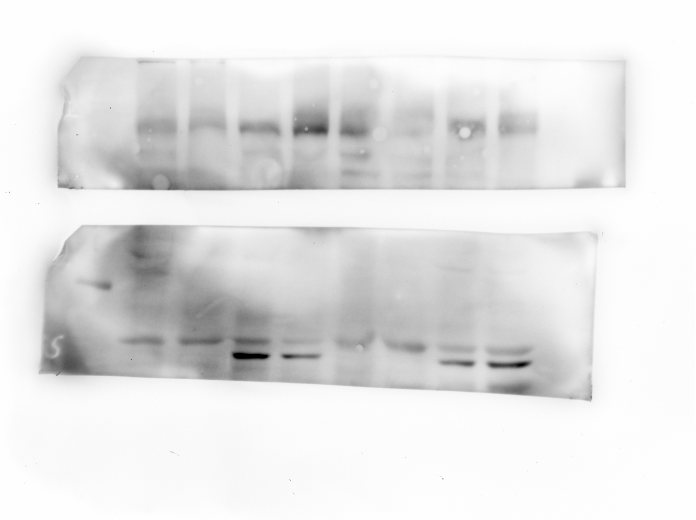

Supplement: Supplementary file 1 [file DataSheet1.ZIP › row images for WB/Figure 3E left HIF2A.tif]

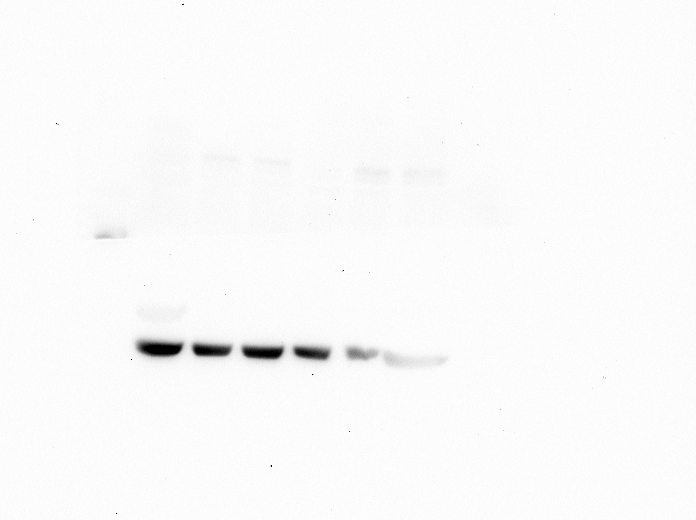

Supplement: Supplementary file 1 [file DataSheet1.ZIP › row images for WB/Figure 3E left tubulin.tif]

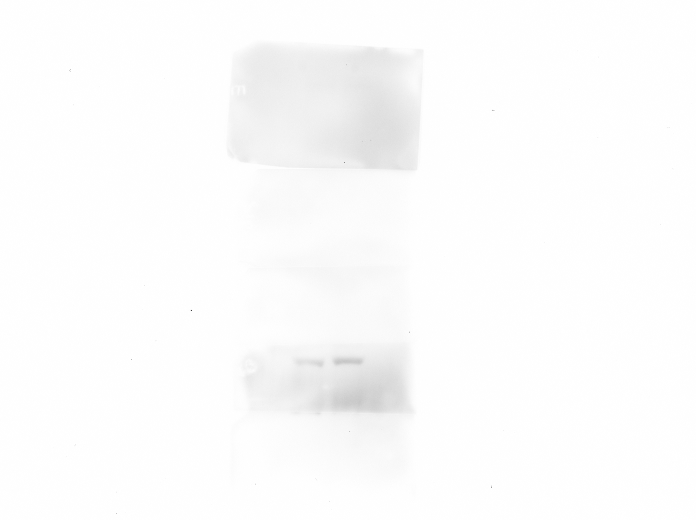

Supplement: Supplementary file 1 [file DataSheet1.ZIP › row images for WB/Figure 3E right HIF2A.tif]

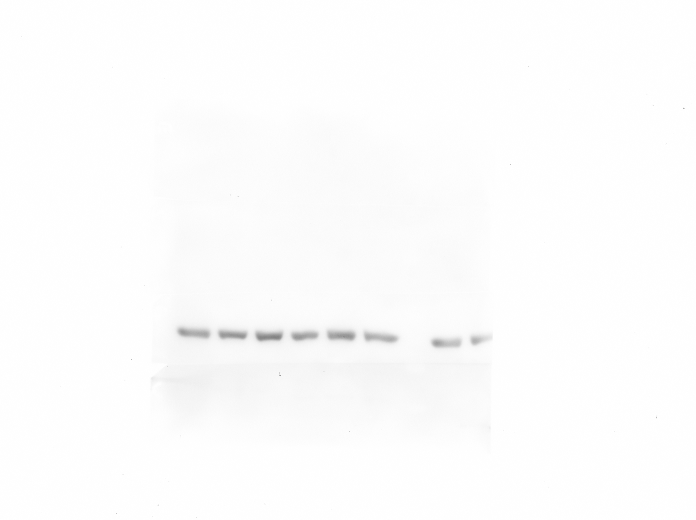

Supplement: Supplementary file 1 [file DataSheet1.ZIP › row images for WB/Figure 3E right tubulin.tif]

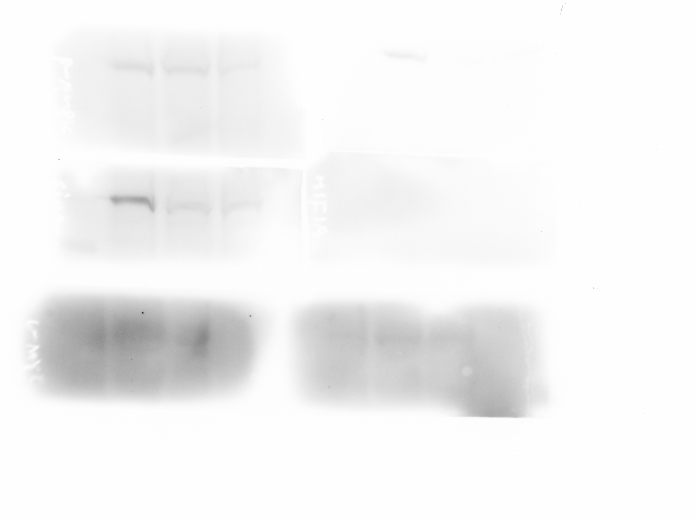

Supplement: Supplementary file 1 [file DataSheet1.ZIP › row images for WB/Figure 4A HIF2A.tif]

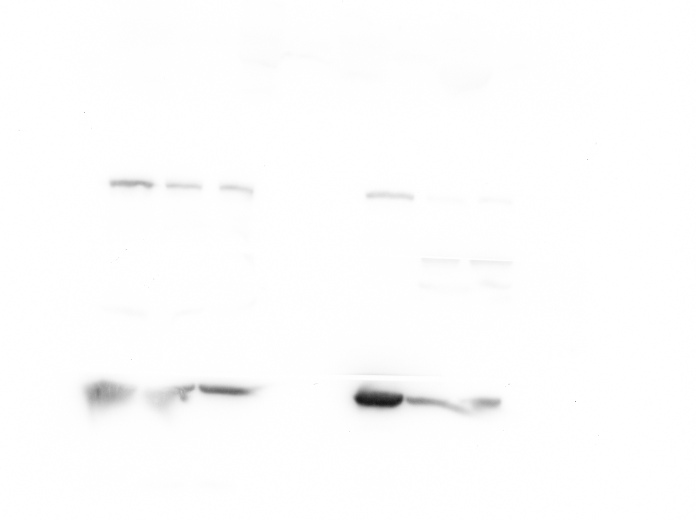

Supplement: Supplementary file 1 [file DataSheet1.ZIP › row images for WB/Figure 4A HK2.tif]

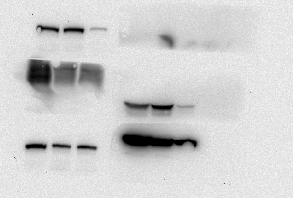

Supplement: Supplementary file 1 [file DataSheet1.ZIP › row images for WB/Figure 4A LDHA.tif]

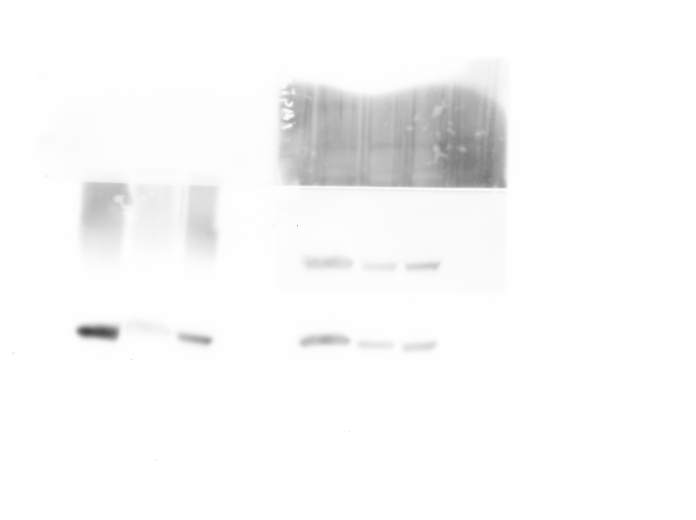

Supplement: Supplementary file 1 [file DataSheet1.ZIP › row images for WB/Figure 4A PDK1.tif]

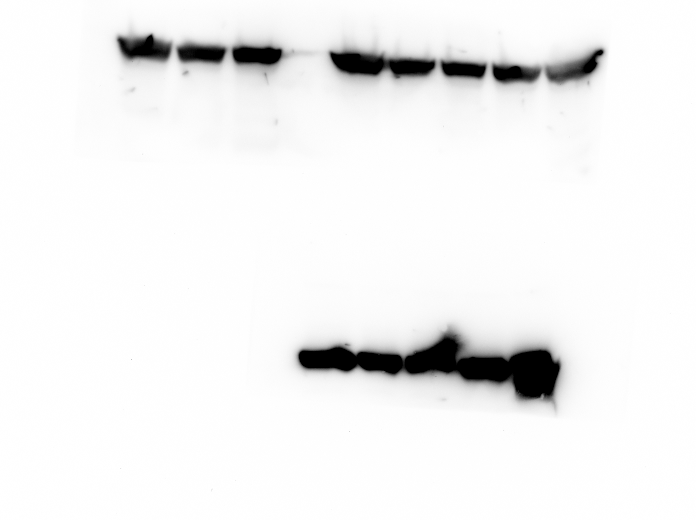

Supplement: Supplementary file 1 [file DataSheet1.ZIP › row images for WB/Figure 4A tubulin.tif]

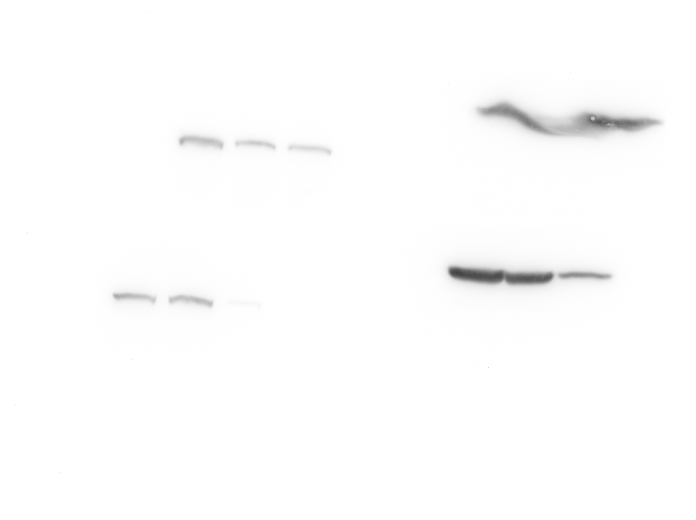

Supplement: Supplementary file 1 [file DataSheet1.ZIP › row images for WB/Figure 4D HK2.tif]

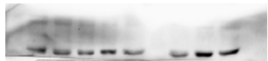

Supplement: Supplementary file 1 [file DataSheet1.ZIP › row images for WB/Figure S1 SIPA1.tif]

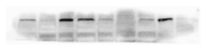

Supplement: Supplementary file 1 [file DataSheet1.ZIP › row images for WB/Figure S2 SIPA1.tif]

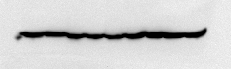

Supplement: Supplementary file 1 [file DataSheet1.ZIP › row images for WB/Figure S2 tubulin.tif]

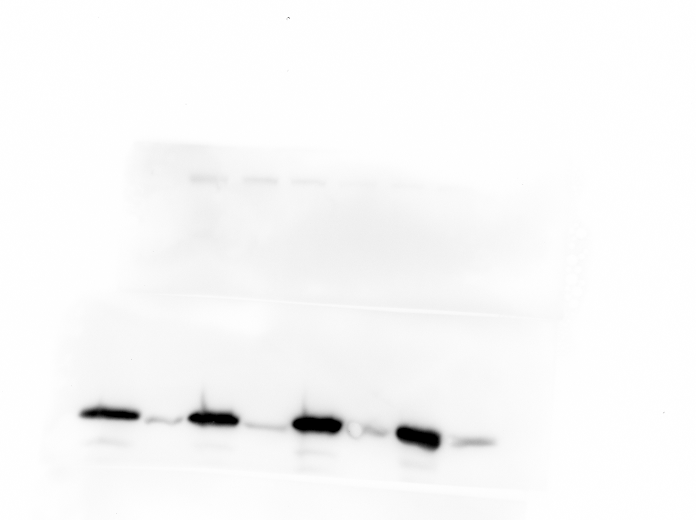

Supplement: Supplementary file 1 [file DataSheet1.ZIP › row images for WB/Figure S4 GAPDH.tif]

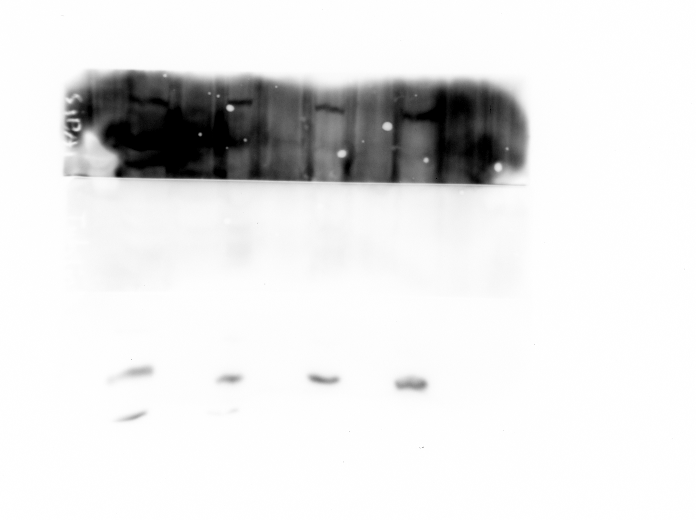

Supplement: Supplementary file 1 [file DataSheet1.ZIP › row images for WB/Figure S4 H2A.tif]

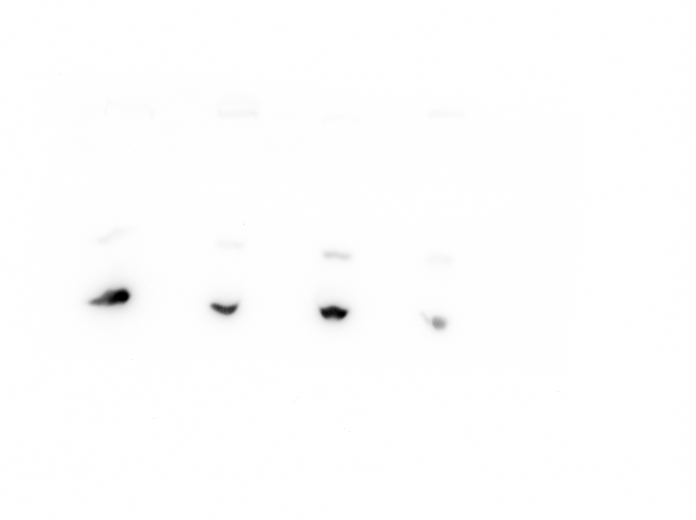

Supplement: Supplementary file 1 [file DataSheet1.ZIP › row images for WB/Figure S4 SIPA1 H2A.tif]
