# Supplementary material for: SIPA1 Enhances Aerobic Glycolysis Through HIF-2α Pathway to Promote Breast Cancer Metastasis
Source: Front Cell Dev Biol. 2022 Jan 12;9:779169. doi: 10.3389/fcell.2021.779169 (PMC8790513; doi:10.3389/fcell.2021.779169)
Supplement: Supplementary file 2 [file DataSheet2.docx]

**Supplementary data**

**Figure S1.**


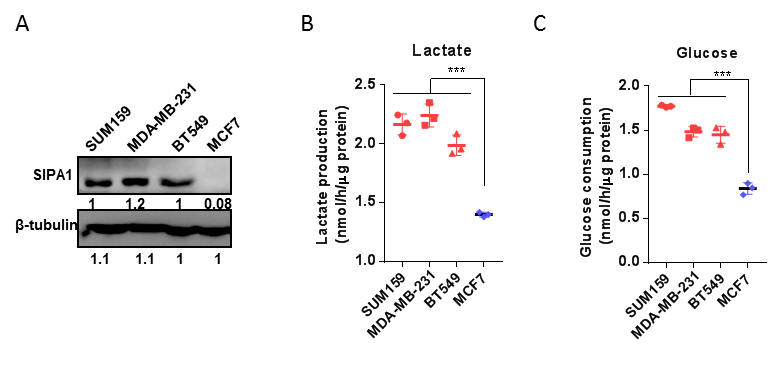


**Figure S1. SIPA1 regulates aerobic glycolysis in breast cancer cell lines.** (A) SIPA1 protein expression levels in four representative breast cancer cell lines of SUMI159, MDA-MB-231, BT549, and MCF7 were determined by Western blotting analysis. β-tubulin was included as a control. Lactate production (B) and glucose consumption (C) in these cells were determined. Data were presented as mean ± s.d. and all the experiments were conducted in triplicate. ****P* < 0.001. (Student’s *t*-test).

**Figure S2**

**Figure S2. SIPA1 regulates aerobic glycolysis in BT549 breast cancer cell lines.** (A) Establishment of BT549 breast cancer cell lines expressing low levels of SIPA1. Wild-type and SIPA1-knockdown BT549 cells were examined for SIPA1 expression levels. β-Tubulin was included as a reference. Lactate production (B) and glucose consumption (C) in these cells were determined. Data were presented as mean ± s.d. of triplicate measurements (n=3). ***P < 0.001. (Student’s t-test).

**Figure S3.**

**Figure S3. Knocking down SIPA1 decreased the transcription of glycolysis- and hypoxia-related genes in BT549 breast cancer cells.** mRNA levels of genes involved in glycolysis and responses to hypoxia in parental and SIPA1-knowckdown BT549 breast cancer cells were detected by qRT-PCR. Data were shown as mean ± s.d. of triplicate measurements (n=3). SDHC was included as an endogenous control.

**Figure S4.**

**Figure S4. SIPA1 was overexpressed in TNBC cells.** Cells lines in GSE41313 dataset were separated into three groups (luminal, Her2+, and TNBC). Expression values of SIPA1 were presented with log2 FPKM.

**Figure S5.**

**Figure S5. Knocking down EPAS1 suppressed the transcription of both glycolysis- and hypoxia-related genes in MDA-MB-231 cells.** mRNA levels of genes involved in glycolysis and responses to hypoxia were determined by qRT-PCR. Data were shown as mean ± s.d. of triplicate measurements (n=3). SDHC was included as an endogenous control.

**Figure S6.**

**Figure S6. Time course of the body weight of xenografted mice.** During the course of oxamate treatment, the bodyweight of mice was measured every day for 14 days. Data were shown as mean ± s.d.. Each group contained 6 mice.

**Table S1. List of oligo shRNA sequences targeted to *SIPA1* or *EPAS1***

| Name | Sequence (5’-3’) |
| --- | --- |
| sh*SIPA1* forward | CCGGTACTACCGCAAATACTTCTATCTCGAGATAGAAGTATTTGCGGTAGTATTTTTG |
| sh*SIPA1* reverse | AATTCAAAAATACTACCGCAAATACTTCTATCTCGAGATAGAAGTATTTGCGGTAGTA |
| sh*EPAS1*-2 forward | CCGGGCTGGAGTATGAAGAGCAACTCGAGTTGCTCTTCATACTCCAGCTTTTTG |
| sh*EPAS1*-2 reverse | AATTCAAAAAGCTGGAGTATGAAGAGCAACTCGAGTTGCTCTTCATACTCCAGC |
| sh*EPAS1*-3 forward | CCGGGCGCAAATGTACCCAATGATACTCGAGTATCATTGGGTACATTTGCGCTTTTTG |
| sh*EPAS1*-3 reverse | AATTCAAAAAGCGCAAATGTACCCAATGATACTCGAGTATCATTGGGTACATTTGCGC |

**Table S2. Primer list for ChIP-PCR**

| Name | Sequence (5’-3’) |
| --- | --- |
| Seg1 forward | TCTCACGACATTGGCCTAAAGC |
| Seg1 reverse | ACTCCATCCAAACGAAACCAGC |
| Seg2 forward | TCAGAGGTCACACTCATCAGCC |
| Seg2 reverse | CAACCAGAAAATCTGGAGTCCC |
| Seg3 forward | TAAGAGACAGGAGCAAGGCATG |
| Seg3 reverse | TGTACCTTCTCCAGCCACTGTC |

**Table S3. Primer list for qRT-PCR**

| Name | Sequence (5’-3’) |
| --- | --- |
| *SLC2A1* forward | CATCCCATGGTTCATCGTGGCTGAACT |
| *SLC2A1* reverse | GAAGTAGGTGAAGATGAAGAACAGAAC |
| *HK2* forward | GCCATCCTGCAACACTTAGGGCTTGAG |
| *HK2* reverse | GTGAGGATGTAGCTTGTAGAGGGTCCC |
| *GPI* forward | TATTGTGTTCACCAAGCTCACACC |
| *GPI* reverse | TGGTAGAAGCGTCGTGAGAGGTC |
| *ALDOA* forward | AGGCCATGCTTGCACTCAGAAGT |
| *ALDOA* reverse | AGGGCCCAGGGCTTCAGCAGG |
| *GAPDH* forward | TTCCGTGTCCCCACTGCCAACGT |
| *GAPDH* reverse | CAAAGGTGGAGGAGTGGGTGTCGC |
| *PGK1* forward | ATGTCGCTTTCTAACAAGCTGA |
| *PGK1* reverse | GCGGAGGTTCTCCAGCA |
| *PGAM1* forward | GGAAACGTGTACTGATTGCAGCCC |
| *PGAM1* reverse | TTCCATGGCTTTGCGCACCGTCT |
| *ENO2* forward | TCATGGTGAGTCATCGCTCAGGAG |
| *ENO2* reverse | ATGTCCGGCAAAGCGAGCTTCATC |
| *PKM2* forward | GCCCGTGAGGCAGAGGCTGC |
| *PKM2* reverse | TGGTGAGGACGATTATGGCCC |
| *LDHA* forward | ATGGCAACTCTAAAGGATCA |
| *LDHA* reverse | GCAACTTGCAGTTCGGGC |
| Tubulin forward | CCAAGCTGGAGTTCTCTA |
| Tubulin reverse | CAATCAGAGTGCTCCAGG |
| *SIPA1* forward | CAGTGGCTCTGAGGACAAGG |
| *SIPA1* reverse | GGGTCTCCACTCTCTGGGAT |
| *PDK1* forward | AGATAATCTTCTCAGGACACCATCC |
| *PDK1* reverse | TATCTGTAAAGATCATCTTGCAGGC |
| *TGFB1* forward | GAGAAGCGGTACCTGAACCC |
| *TGFB1* reverse | TGAACCCGTTGATGTCCACT |
| *VEGFA* forward | AGGCCAGCACATAGGAGAGA |
| *VEGFA* reverse | ACGCGAGTCTGTGTTTTTGC |
| *EPAS1* forward | TGACAGCTGACAAGGAGAAGAA |
| *EPAS1* reverse | TGTGTTCGCAGGAAGCTGAT |
| *CA9* forward | TAGCCCTGGTTTTTGGCCTC |
| *CA9* reverse | GTAGCTCACACCCCCTTTGG |
